# BPSDiary study protocol: a multi-center randomized controlled trial to compare the efficacy of a BPSD diary vs. standard care in reducing caregiver's burden

**DOI:** 10.3389/frdem.2023.1301280

**Published:** 2023-12-18

**Authors:** Federico Emanuele Pozzi, Luisa Calì, Fabrizia D'Antonio, Arianna Ida Altomare, Micaela Sepe Monti, Massimiliano Panigutti, Adolfo Di Crosta, Rocco Palumbo, Laura Bonanni, Valentina Carlucci, Cinzia Bussè, Annachiara Cagning, Daniele Urso, Davide Vilella, Giancarlo Logroscino, Margherita Alberoni, Angelo Bellinvia, Elisabetta Farina, Francesca de Rino, Armando Gavazzi, Marta Zuffi, Giuseppe Bruno, Valentina Bessi, Matteo Cotta Ramusino, Giulia Perini, Alfredo Costa, Carlo Ferrarese, Ildebrando Appollonio, Lucio Tremolizzo

**Affiliations:** ^1^Neurology Department, Fondazione IRCCS San Gerardo dei Tintori, Monza, Italy; ^2^Milan Center for Neuroscience (NeuroMI), University of Milano-Bicocca, Milan, Italy; ^3^School of Medicine and Surgery, University of Milano-Bicocca, Milan, Italy; ^4^Human Neuroscience Department, Sapienza Università di Roma, Rome, Italy; ^5^Neuropsychology Unit, IRCCS Fondazione Santa Lucia, Rome, Italy; ^6^Psychiatry Unit, Department of Health Sciences, University of Florence, Florence, Italy; ^7^Department of Medicine and Aging Sciences, University G. d'Annunzio of Chieti-Pescara, Chieti, Italy; ^8^Neurology Unit, Department of Neuroscience, University of Padova, Padua, Italy; ^9^Center for Neurodegenerative Diseases and the Aging Brain, Department of Clinical Research in Neurology, University of Bari Aldo Moro, “Pia Fondazione Cardinale G. Panico”, Lecce, Italy; ^10^Neurology Department, IRCCS Fondazione Don Carlo Gnocchi, Milan, Italy; ^11^Neurology Department, MultiMedica Castellanza, Castellanza, Italy; ^12^Department of Neuroscience, Psychology, Drug Research and Child Health, University of Florence, Florence, Italy; ^13^Research and Innovation Centre for Dementia-CRIDEM, AOU Careggi, Florence, Italy; ^14^Unit of Behavioral Neurology Fondazione IRCCS Mondino, and University of Pavia, Pavia, Italy

**Keywords:** BPSD, dementia, protocol, diary, behavior

## Abstract

Behavioral and Psychological Symptoms of Dementia (BPSD) are a heterogeneous set of psychological and behavioral abnormalities seen in persons with dementia (PwD), significantly impacting their quality of life and that of their caregivers. Current assessment tools, such as the Neuropsychiatric Inventory (NPI), are limited by recall bias and lack of direct observation. This study aims to overcome this limitation by making caregiver reports more objective through the use of a novel instrument, referred to as the BPSDiary. This randomized controlled trial will involve 300 caregiver-PwD dyads. The objective is to evaluate whether the use of the BPSDiary could significantly reduce caregiver burden, assessed using the Zarit Burden Interview (ZBI), compared to usual care. The study will include adult PwD, caregivers living with or close to the patient, and BPSD related to the HIDA (hyperactivity, impulsivity, irritability, disinhibition, aggression, agitation) domain. Caregivers randomized to the intervention arm will use the BPSDiary to record specific BPSD, including insomnia, agitation/anxiety, aggression, purposeless motor behavior, and delusions/hallucinations, registering time of onset, severity, and potential triggers. The primary outcome will be the change in ZBI scores at 3 months, with secondary outcomes including changes in NPI scores, olanzapine equivalents, NPI-distress scores related to specific BPSD domains, and caregiver and physician satisfaction. The study will be conducted in 9 Italian centers, representing diverse geographic and sociocultural contexts. While potential limitations include the relatively short observation period and the focus on specific BPSD disturbances, the BPSDiary could provide physicians with objective data to tailor appropriate non-pharmacological and pharmacological interventions. Additionally, it may empower caregivers by encouraging reflection on BPSD triggers, with the potential to improve the quality of life for both PwD and their caregivers.

**Trial registry:**

NCT05977855.

## 1 Introduction

The behavioral and psychological symptoms of dementia (BPSD) are a heterogeneous set of psychological reactions, psychiatric symptoms, and behavioral abnormalities that are present in persons with dementia (PwD) due to any etiology (D'antonio et al., 2022). These have been grouped in different clusters across different studies. One of the most common classifications divides them into five clusters: psychosis (delusions and hallucinations), affective symptoms (anxiety and depression), apathy, psychomotor symptoms (irritability, aberrant motor behaviors, agitation), and mania (euphoria and disinhibition). In Italy, the most common BPSD seem to be agitation, apathy, depression, psychosis, and aggression (D'antonio et al., 2022). It is likely that cultural and ethnic differences might partially explain the heterogeneity and relevance of BPSD across different countries and settings, as well as caregivers' responses (Abe et al., 2015).

BPSD are extremely relevant for PwD, as they are associated with a faster cognitive decline, loss of independence, falls, and institutionalization (Calsolaro et al., 2021). BPSD might be present across all stages of dementia, and even predate it (in the so-called mild behavioral impairment, MBI) (Ismail et al., 2017). Specific BPSD, such as apathy, might also predict conversion from mild cognitive impairment to dementia (Fresnais et al., 2022).

Several instruments have been proposed to assess BPSD. More than sixty scales and tools have been developed, the most commonly used probably being the Neuropsychiatric Inventory (NPI); however, none of them seems to satisfactorily address all relevant aspects of BPSD (Pozzi et al., 2023). Most instruments are limited by the high degree of recall bias, lack of direct observation, and length (D'antonio et al., 2022). Finally, an intrinsic risk using broad tools such as the NPI is the imprecise evaluation of BPSD as a unitary construct, aggregating symptoms with different biological basis and treatments (Cho et al., 2021).

Several modifiable and non-modifiable risk factors for BPSD have been identified in the literature. Inconclusive results have been published for APOE4, while polymorphisms in other genes, such as COMT, serotonin receptor 2A and IL-1β seem to correlate with specific BPSD (Flirski et al., 2011). Other non-modifiable “background factors” might be low education and female gender (Chang et al., 2020). On the contrary, “proximal factors,” which are temporally close to BPSD, might be potentially modifiable, and mostly include triggers and relational aspects between PwD and their caregivers (Cho et al., 2021; Nagata et al., 2022).

Current guidelines suggest to address proximal factors with non-pharmacological strategies as a first line treatment (Calsolaro et al., 2021). In practice, psychotropic medication are still largely used, possibly with the exception of BPSD pertaining to eating and sleeping (D'antonio et al., 2022). However, antipsychotics have several side effects (D'antonio et al., 2022), and despite FDA and European Medicine Agency black-box warnings they are often used for long periods, with an increasing risk of parkinsonism, sedation, falls, stroke, cognitive decline and death (Calsolaro et al., 2021). Nevertheless, the use of antipsychotics, acetylcholinesterase inhibitors and memantine seems to be associated with a reduced caregiver burden (Levy et al., 2012).

Non-pharmacological algorithms include DICE (Describe, Investigate, Create, Evaluate) (Kales et al., 2019) and DATE (Describe & Measure, Analyze, Treat, Evaluate) (Cho et al., 2021). Both rely on precise characterization of triggers to find optimal solution to address BPSD. Functional analysis is generally based on ABC approach, evaluating antecedent (trigger), behavior description and consequence. A meta-analysis demonstrated that functional analysis with tailored strategies for PwD and caregivers positively impact burdensome BPSD frequency and caregivers' reaction, but has no effect on incidence or severity of BPSD, mood or caregiver burden (Moniz Cook et al., 2008). In Italy, caregiver education is the most common non-pharmacological intervention, used by 86% of caregivers as a fist line (D'antonio et al., 2022).

An unmet need for physicians is the availably of more objective tools for severity, frequency and context of BPSD, in order to apply non-pharmacological strategies (Loi and Lautenschlager, 2017). In Italy, 93% of physicians working in the field of dementia would be interested in a new tool to address BPSD, which reflects the unsatisfactory nature of current options (D'antonio et al., 2022).

Among BPSD, those belonging to the HIDA domain (hyperactivity, impulsivity, irritability, disinhibition, aggression, agitation) seem to be the most difficult to treat (Van der Linde et al., 2014; Keszycki et al., 2019). Person-centered strategies, including positive and significant social interactions, reminiscence therapy among others, seem to have a beneficial effect, albeit modest (Keszycki et al., 2019). A recent study evaluated caregiver burden and BPSD assessment with daily phone interviews over the course of eight days, showing that the quality of the relationship between caregivers and PwD attenuates caregivers stress related to BPSD (Chunga et al., 2021). Several instruments exist to evaluate caregiver burden, the most common being the Zarit Burden Interview (ZBI) (Melo et al., 2011; Liu et al., 2017; Griffiths et al., 2018; Evans et al., 2021; Jhang et al., 2021; Kanemoto et al., 2021), whose Italian version has been validated and is freely available (Chattat et al., 2011). The ZBI includes 22 questions, each scored with a 0–4 Likert scale, with a total score above 25 indicating a clinically significant burden. Generally speaking, the most burdensome BPSD include delusions, aggression, aberrant motor behavior, agitation and irritability, which substantially overlap with the HIDA domain (Fauth and Gibbons, 2014; Hiyoshi-Taniguchi et al., 2018).

A rather neglected field is satisfaction of caregivers for care provided by physicians to PwD. No instrument for healthcare satisfaction has been validated in caregivers of PwD, or in BPSD, and many instruments seem excessively long.^1^ On the other hand, there is a lack of instruments evaluating physicians satisfaction in treating PwD.

The main idea of our study is to make caregiver's evaluation of BPSD more objective, partially eliminating recall bias through the use of a daily diary focusing on the most disturbing BPSD and their triggers. This would also overcome the limitation of the observation by the physician, which is limited to the short period of the visits and may not capture relevant phenomena present at home. A diary has already been used in another recent study, with a checklist based on NPI in one case (Cho et al., 2021), and a rather long checklist of 53 behaviors in another one (Fauth et al., 2006). However, none of these approaches was treatment-oriented, and triggers were not evaluated. Therefore, we expect that our instrument, called “BPSDiary,” would allow a more precise and treatment-oriented assessment of BPSD, providing physicians with relevant data to implement non-pharmacological and tailored strategies to address them and eventually reduce caregivers' burden.

## 2 Methods and analysis

Based on the premises mentioned above, we present here the protocol for a non-pharmacological parallel-arm randomized controlled trial named “Use of a Diary to Assess and Monitor Behavioral and Psychological Symptoms of Dementia.” The study will randomize 300 dyads (persons with dementia and their caregivers) to either the use of the BPSDiary to record BPSD or usual care. In this context, usual care means the way involved physicians usually treat their patients, according to existing guidelines, personal experience, or both. The main objective is to evaluate whether the use of the BPSDiary can lead to a significant reduction of ZBI scores at 3 months compared to usual care.

Inclusion criteria will be as follows:

- Adult patients with cognitive impairment (either MCI or dementia, either neurodegenerative or vascular or both, diagnosed by a physician with experience in dementia care)
- Willingness of both caregiver and patient to take part in the study
- Caregiver living with the patient, or able to cover the whole day (e.g., caregiver living in the same building)
- Presence of BPSD pertaining to the HIDA domain, as inferred by the physician during the screening visit
- Signed informed consent from both patient and caregiver, as requested by the ethical committee

Dyads will be excluded if they do not provide written consent to participate in the study.

**Figure 1:**
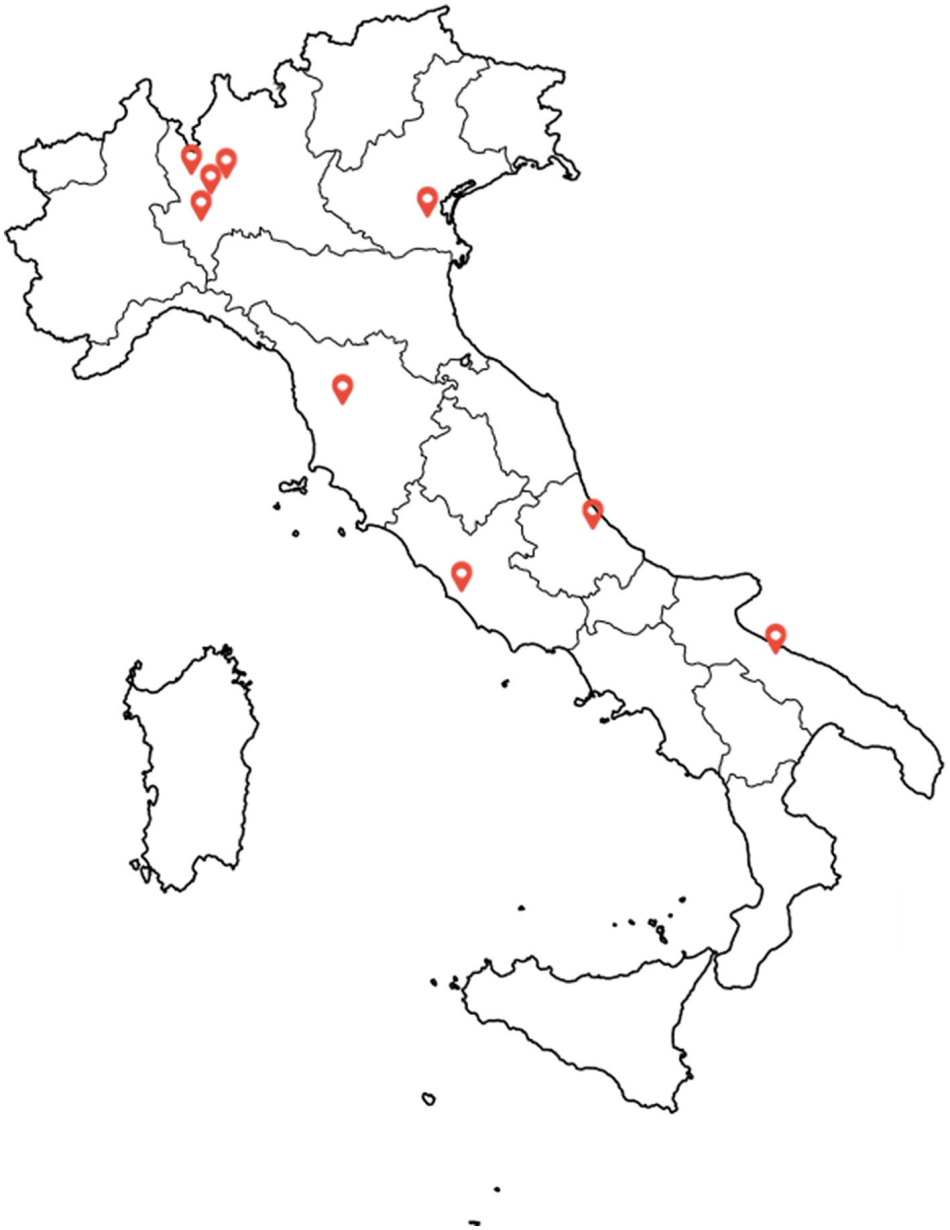
Geographical distribution of involved centers. From North to South: Castellanza (west), Monza (east), Milan, Pavia, Padua (east), Florence, Chieti, Rome, Bari.

The study will be conducted across the Centers for Dementia and Cognitive Decline of nine Italian centers, distributed along the peninsula. These centers have been selected based on their regional importance and expertise in managing BPSD. Sixty patients will be recruited at the Fondazione IRCCS San Gerardo dei Tintori (Monza, Lombardy), while 30 patients each will be enrolled in the remaining centers. These will include the following eight centers: Fondazione IRCCS Don Gnocchi (Milan, Italy), IRCCS Fondazione Mondino (Pavia, Italy), Ospedale Multimedica (Castellanza, Lombardy), AO Padova (Padova, Veneto), AOU Careggi (Florence, Tuscany), Sapienza University (Rome, Lazio), Ospedale SS Annunziata (Chieti, Abruzzo), Pia Fondazione Cardinale Panico (Bari, Apulia). The geographical distribution of the involved centers is shown in Figure 1.

The dyads will be randomized in a 1:1 allocation ratio with a process of covariate adaptive randomization (Suresh, 2011), using caregiver age (cut-off 60 years old; this cut-off is chosen to ensure an equal representation of young/middle-age and elderly caregivers), patient comorbidity [Cumulative Illness Rating Scale (CIRS) – comorbidity index (Salvi et al., 2008) cut-off of 1], patient gender, patient functional independence [Activities of Daily Living (ADL) and Instrumental Activities of Daily Living (IADL) preserved vs. either of them not preserved] and baseline ZBI (cut-off of 31 points). Randomization will be performed through a dedicated software, as previously shown by other groups (O'Callaghan, 2014; Guillaumes and O'Callaghan, 2019).

At baseline, the following scales will be administered to the dyads: CIRS, ADL, IADL, NPI, ZBI, MMSE. These scales will be administered in a quiet environment, free of distractions. Data will also be gathered on age, gender, education of both caregivers and patients, and drugs used by the patients.

The caregivers randomized to the intervention arm will be instructed to use the BPSDiary to register specific behaviors of interest (the BPSDiary is available in the Supplementary material). These include insomnia, agitation/anxiety, aggression (either physical or verbal), purposeless motor behavior, delusions/hallucinations. The caregiver will have to mark any occurrence of these BPSD by indicating the time of onset, ticking the appropriate severity box (mild or severe) and writing down possible triggers. An introductory page explains the definitions of each term in plain language and provides examples. The data in the diary are then transposed on an interactive excel file (available at https://drive.google.com/file/d/1i3_NY7MRg9uoe2kD5njkUhax1zS9engp/view?usp=sharing) that allows the treating physician to analyze several aspects of the BPSD of the patient, such as their temporal pattern, severity, prevalence and triggers. The BPSDiary is only offered in a “paper and pencil” version, to avoid issues related with poor digital skills of elderly caregivers in Italy.

After the first 6 weeks the caregiver will be contacted by telephone and he/she will be asked if there are any issues related to BPSD. Caregivers randomized to the intervention arm will be asked to send the first six pages of the diary for analysis. Appropriate actions will be taken to address any issue according to the treating physician's judgment; options may include telephone counseling, scheduling a visit, prescribing or deprescribing drugs and so on. All of this will be repeated after other 6 weeks. All the approaches will be recorded and accounted for in the analyses.

The following scales will be re-administered at the end of the study (3 months): NPI, ZBI, and the satisfaction questionnaires (available in the Supplementary material). The other scales (ADL, IADL, MMSE) will not be administered, as they are judged unlikely to significantly change in the study period, and even if they do, this would probably not be related to the use of the diary.

The caregiver will be allowed to contact the treating physician at any time during the entirety of the study in case urgent actions need to be taken to address BPSD; dyads randomized to the diary will be asked to send it to the doctor for analysis. Actions taken during these unscheduled contacts will be registered in the following scheduled contact window (e.g., if the contact takes place at week 4 and quetiapine is introduced, this will be registered at the first of the two scheduled contacts at week 6).

The primary outcome will be the variation on the ZBI at 3 months. Secondary outcomes will include variation on NPI, olanzapine equivalents [calculated with the Defined Daily Doses method (Leucht et al., 2016)], caregiver care-related satisfaction, and variation on relevant NPI-distress scores at 3 months, as well as caregiver and physician diary-related satisfaction. Relevant NPI-distress scores include scores related to insomnia, agitation, aberrant motor behavior, aggression, delusions, hallucinations (i.e., the domains assessed in the BPSDiary).

The entire study will last 13 months. Enrollment will take place over the course of 9 months, and each dyad will be followed for 12 weeks. Data analysis is expected to take another month.

**Figure 2:**
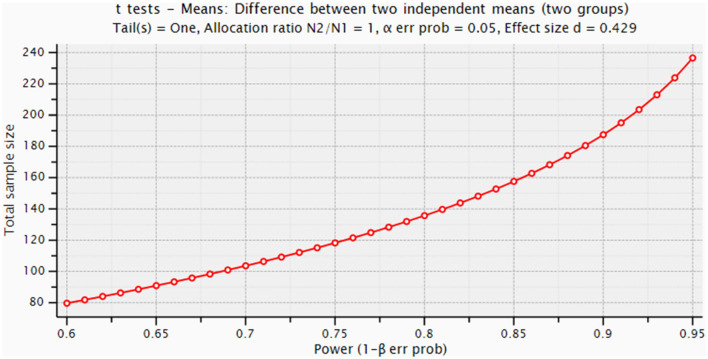
Power graph for sample size calculation.

Sample size has been computed with the software gpower (Erdfelder et al., 2009) considering a clinically significant difference of at least 6 points at the ZBI, corresponding to an effect size of 0.428 with a *t*-test. Considering α = 0.05 and β = 0.05, 238 would be required. We hypothesized a drop-out rate of 20%, based on clinical experience on similar studies; therefore, 300 dyads are required. The power graph is shown in Figure 2.

A *t*-test will be used to compare differences in ZBI variations (ZBI_followup_-ZBI_baseline_) between the two arms. A *t*-test will be also used to compare differences between the two arms in NPI, NPI-distress, olanzapine equivalents variations, as well as satisfaction questionnaires scores. Pre-specified analyses will include linear mixed effect models to compare ZBI trajectories, including arm, person's and caregiver's age, baseline MMSE, person's and caregiver's education, and olanzapine equivalents as possible predictors.

The registration data set is reported in Table 1. The CONSORT checklist (Schulz et al., 2010) for this RCT is available in the Supplementary material.

**Table 1:** Registration data set.

**Data category**	**Information**
Primary registry and identifying number	ClinicalTrials.gov, NCT05977855
Date of registration	August 7^th^, 2023
Primary sponsor	University of Milano-Bicocca
Sources of monetary or material support	University of Milano-Bicocca
Contact for public or scientific queries	Federico Emanuele Pozzi, MD [federicoemanuele.pozzi@gmail.com], Fondazione IRCCS San Gerardo dei Tintori, Monza, Italy
Scientific title	Use of a diary to assess and manage Behavioral and Psychological Symptoms of Dementia [BPSDiary]
Countries of recruitment	Italy
Health condition or problem studied	Behavioral and psychological symptoms of dementia
Intervention	Diary to record five different BPSD vs. Standard of Care
Key inclusion and exclusion criteria	Inclusion criteria: - Neurodegenerative or vascular cognitive impairment (MCI or dementia) - Willingness of both caregiver and patient to take part in the study - Caregiver living with the patient, or able to cover the whole day - BPSD pertaining to the HIDA domain - Signed informed consent • Exclusion criteria: - Refusal to take part in the study
Study type	Interventional, non-pharmacological • Allocation: randomized • Masking: none • Primary purpose: treatment
Planned begin of enrollment	September 2023
Target sample size	300
Recruitment status	Recruiting
Primary outcome	ZBI variation at 3 months
Key secondary outcomes	- NPI variation - Olanzapine equivalents variation - Caregiver care-related satisfaction - NPI-distress variations^*^ - Caregiver and physician diary-related satisfaction

HIDA, Hyperactivity, impulsivity, irritability, disinhibition, aggression, agitation; NPI, Neuropsychiatric Inventory; ZBI, Zarit Burden Interview. ^*^only pertaining to items included in the diary.

## 3 Discussion

Several guidelines suggest the use of non-pharmacological interventions as a first line in the treatment of BPSD. However, the use of antipsychotics and other psychoactive medications is still common in Italy (Azermai et al., 2013), and this might be dependent on the fact that addressing triggers of disruptive behaviors is hard and heavily influenced by recall bias. Moreover, it seems that caregivers perceive systemic barriers in non-pharmacological interventions which are partially dependent on the inability of physicians to provide information, support, and coaching about such strategies (Kerns et al., 2018). On the contrary, caregivers might have the wrong idea that psychotropic medications are generally safe and effective, as they may not be aware of FDA black-box warnings on antipsychotics (Kerns et al., 2018). The use of the BPSDiary could theoretically allow for the identification of such triggers, which would help the clinician finding tailored non-pharmacological solutions for the management of BPSD. Moreover, it could allow caregivers to reflect on the mechanisms and causes of behaviors, which might give them a sense of empowerment.

A strength of our protocol is the representation of almost all Italian peninsula. Thanks to the variety of customs and societal characteristics in different Italian regions, the inclusion of centers representative of Northern, Central, and Southern Italy, as well as serving both Italian biggest cities (such as Rome and Milan) and smaller realities (such as Castellanza) will allow exploring the use of the diary in diverse settings, drawing considerations about its reception, adaptability, and implementation. This is important, as several disparities among Italian regions and contexts, such as living conditions, education, and social isolation, which are associated with varying levels of reported health (Franzini and Giannoni, 2010), may also influence the assessment and management of BPSD. Another strength is the large anticipated sample size, that will incidentally allow exploring secondary outcomes, as well as analyzing the effect of different variables such as specific dementia type to evaluate which kind of patient might benefit the most from the intervention.

We acknowledge that the inclusion of people with MCI might be controversial. On one hand, MCI people are usually not expected to be cared for by a caregiver, as their level of cognitive impairment should not significantly impact their daily activities by definition. On the other hand, we cannot exclude the fact that a caregiver might be indeed present for elderly people even when their cognitive impairment is still mild, especially when there are other comorbidities. Perhaps inherently to Italian society, it is still common to provide “care” for elderly relatives even when there is no loss of autonomy. Moreover, certain behavioral abnormalities might be indeed present in the early phases of certain neurodegenerative conditions such as DLB even in the “MCI” phase. For instance, the presence of hallucinations is a core feature of the 2020 McKeith's criteria of MCI-LB (McKeith et al., 2020). In other cases, MCI might be associated with a condition called mild behavioral impairment, which may increase the likelihood of progression of the cognitive impairment (Mallo et al., 2018; Ruthirakuhan et al., 2022).

A foreseeable limitation is the relatively short period of observation. However, this is comparable with the observation period used in other tools we used to evaluate concurrent validity, such as the NPI (Kaufer et al., 1998; Pozzi et al., 2023). Moreover, the inclusion criteria imply the presence of BPSD relative to the HIDA domain, which are associated with worse outcomes in PwD. Therefore, longer periods of observation could in theory increase the drop-out rate and therefore require an even larger sample size. A period of 3 months was judged to be a good compromise between all these considerations.

Another potential weakness is the decision to restrict BPSD assessment to only a certain subset of symptoms. Broad BPSD assessment tools that evaluate all possible disturbances already exist (Kales et al., 2017, 2018), but our intention was to target specifically those symptoms that greatly affect caregivers and result in worse outcomes for the PwD (Fauth and Gibbons, 2014; Hiyoshi-Taniguchi et al., 2018). On one hand, this will ensure that our tool is easy to use and understand, requiring caregivers to detect only the most striking behaviors, and also fast to complete, requiring only a minimum set of information to be provided. The decision to distinguish only two categories of severity (“mild” and “severe”) is in line with the perceived difficulty of caregiver to assign numbers to quantify behavior. As people of science, we tend to believe that numbers mean everything, but most of caregivers do not categorize their experiences mathematically, and forcing them to do so may result in some extent of arbitrariness that might jeopardize the assessment. We believe that our diary represents a good compromise between the two opposite tendencies of scientists and healthcare users, asking them to provide easy categories for analysis, but at the same time allowing them to reflect on and express freely their understanding of the triggers of BPSD of the PwD that they care for. While we did not conduct a proper feasibility study, during the preparation for the trial we proposed the instrument to 20 dyads at the Fondazione IRCCS San Gerardo and asked them about their opinions (qualitative data not shown). As expected, caregivers who were not cohabitating with patients reported greater difficulty in completing the diary, which led us to require cohabitation or ability to cover the whole day among inclusion criteria.

Finally, it could be argued that a “paper and pencil” diary could be somehow anachronistic in our hyper-digitalized society. However, our choice was mainly motivated by the fact that most of the caregiver that we expect to recruit will be quite old (i.e., spouses of enrolled PwD). While the creation of apps for senior citizens requires particular care regarding usability issues (Tajudeen et al., 2022), it is also possible that even a perfect app could fail in theory, due to specific country-related issues. Indeed, elderly Italian seem to have restricted digital skills compared to other countries (Caliandro et al., 2021; Melchior, 2023; Vainieri et al., 2023; Valokivi et al., 2023), which could represent a foreseeable barrier for the implementation of a study with an app. Therefore, we expect the paper version of the diary to be more acceptable by the majority of enrolled caregivers. Nevertheless, if the study will show promising results, it is our intention to try and develop an app version, possibly targeting younger caregivers.

## Ethics statement

The study was approved by the Ethical Committee “Comitato Etico Brianza,” with approval number 4251, on May 15^th^, 2023. Recruitment strategies will use different channels. First, the study will be proposed to PwD and caregiver meeting inclusion criteria and already attending the involved memory clinics. Secondly, the study will be advertised on the websites of the involved centers. Lastly, depending on local availability, the study could be advertised to a broader audience through coverage by local media (e.g., newspaper articles).

## Author contributions

FP: Conceptualization, Writing—original draft, Writing—review & editing. LC: Writing—review & editing. FD'A: Writing— review & editing. AA: Writing—review & editing. MS: Writing—review & editing. MP: Writing—review & editing. AD: Writing—review & editing. RP: Writing—review & editing. LB: Writing—review & editing. VC: Writing—review & editing. CB: Writing—review & editing. ACa: Writing—review & editing. DU: Writing—review & editing. DV: Writing—review & editing. GL: Writing—review & editing. MA: Writing—review & editing. BA: Writing—review & editing. EF: Writing—review & editing. FR: Writing—review & editing. AG: Writing—review & editing. MZ: Writing—review & editing. GB: Writing—review & editing. VB: Writing—review & editing. MC: Writing—review & editing. GP: Writing—review & editing. ACo: Writing—review & editing. CF: Writing—review & editing. IA: Writing—review & editing. LT: Writing—review & editing.

## Footnotes

^1^ http://www.psychiatryonline.it/node/3704
