# Supplementary material for: BPSDiary study protocol: a multi-center randomized controlled trial to compare the efficacy of a BPSD diary vs. standard care in reducing caregiver's burden
Source: Front Dement. 2023 Dec 18;2:1301280. doi: 10.3389/frdem.2023.1301280 (PMC11285609; doi:10.3389/frdem.2023.1301280)
Supplement: Supplementary file 2 [file Table_2.DOC]

**Healthcare-associated satisfaction - BPSDiary**

**Caregiver version**

The following questions reflect the degree of your satisfaction with the care provided to the person during the study period (last three months).

Please mark your agreement with each affirmation. There are no wrong or right answers.

We thank you in advance for your collaboration, and we remind you that it is important that you try to answer all the questions.

Name: Surname: Caregiver of: Date:

|  | **Not at all** | **A little** | **Neutral** | **Yes** | **Very** |
| --- | --- | --- | --- | --- | --- |
| 1. Are you *generally* satisfied with the care provided at the memory clinic? | 0 | 1 | 2 | 3 | 4 |
| 1. Are you satisfied with the *management* of behavioral disturbance of the person you care for? | 0 | 1 | 2 | 3 | 4 |
| 1. Are you satisfied with the *communication with the physicians* regarding behavioral disturbances? | 0 | 1 | 2 | 3 | 4 |
| 1. Are you satisfied with *your ability to detect* the behavioral disturbances of the person you care for? | 0 | 1 | 2 | 3 | 4 |
| Only for the BPSDiary group | **Not at all** | **A little** | **Neutral** | **Yes** | **Very** |
| 1. Was it *easy* to use the tool? | 0 | 1 | 2 | 3 | 4 |
| 1. Has the tool *improved your understanding* of the disturbances and their causes? | 0 | 1 | 2 | 3 | 4 |
| 1. Has the tool *improved communication* with the doctor regarding the disturbances? | 0 | 1 | 2 | 3 | 4 |
| 1. Would you like to *keep using* the tool even after the study period? | 0 | 1 | 2 | 3 | 4 |

***THANK YOU FOR YOUR COLLABORATION***

**Healthcare-associated satisfaction - BPSDiary**

**Physician version**

The following questions reflect the degree of your satisfaction with the care provided to the person during the study period (last three months).

Please mark your agreement with each affirmation. There are no wrong or right answers.

We thank you in advance for your collaboration.

Name: Surname: Assisted person: Date:

|  | **Not at all** | **A little** | **Neutral** | **Yes** | **Very** |
| --- | --- | --- | --- | --- | --- |
| 1. Was it *easy* to use the data obtained from the tool? | 0 | 1 | 2 | 3 | 4 |
| 1. Has the tool *improved your understanding* of BPSD and their causes? | 0 | 1 | 2 | 3 | 4 |
| 1. Has the tool *improved communication* with the person and/or caregiver regarding the disturbances? | 0 | 1 | 2 | 3 | 4 |
| 1. Would you like to *keep using* the tool even after the study period for this person? | 0 | 1 | 2 | 3 | 4 |
| 1. Has the tool *improved the management* of the person’s disturbances? | 0 | 1 | 2 | 3 | 4 |
| 1. Has the tool improved the *ability to detect* BPSD in the person? | 0 | 1 | 2 | 3 | 4 |
| 1. Are you *generally* satisfied with the use of the tool with the person? | 0 | 1 | 2 | 3 | 4 |
| 1. Is the tool *more useful* than other scales used to assess and monitor BPSD for the person? | 0 | 1 | 2 | 3 | 4 |
| 1. Has the *caregiver understood* how to correctly use the tool? | 0 | 1 | 2 | 3 | 4 |

***THANK YOU FOR YOUR COLLABORATION***
