# Supplementary material for: BPSDiary study protocol: a multi-center randomized controlled trial to compare the efficacy of a BPSD diary vs. standard care in reducing caregiver's burden
Source: Front Dement. 2023 Dec 18;2:1301280. doi: 10.3389/frdem.2023.1301280 (PMC11285609; doi:10.3389/frdem.2023.1301280)
Supplement: Supplementary file 3 [file Data_Sheet_1.docx]

**BPSDiary**

Pazient: _________________________________ born ___/___/____

Caregiver: _________________________________ born ___/___/____

Relationship with the patient: _________________________________

Start date: ___/___/____

**Introduction**

**Purpose of the BPSDiary**

This diary is a tool to assess behavioral and psychological disturbances associated with dementia. It is a way of making communication with the treating doctor more objective, as well as suggesting effective strategies to manage such problems.

Please fill the diary as completely as possible, recording every day the possible occurrence of the disturbances, their time, severity and potential triggers. To establish the specific disturbance category, please refer to the definitions below. If you are in doubt, please consult the specialist who gave you the diary.

**Definitions:**

**Insomnia or fragmented sleep:** the person cannot sleep, wakes up often during the night, wakes up too early in the morning or do,zes off frequently during the day; or the person is awake, wanders through the house at night, dresses up and undresses, disturbs their relatives’ sleep. This does not apply to the case in which the person wakes up to go to the bathroom 2-3 times per night and then goes back to bed and sleeps immediately.

**Agitation:** the person is intolerant, cranky, whimsical, impatient, does not tolerate delays or waiting for appointments.

**Anxiety:** the person is very nervous, alarmed, frightened without apparent reasons, looks very tense or agitated, or is scared to be separated from their caregiver.

**Physical and verbal aggression:** the person physically and/or verbally rebels to help and assistance, or insults or hits relatives or other people without a proportionate cause.

**Purposeless motor behavior:** the person engages in repetitive or purposeless activities, such as wandering around the house, undressing, untying shoes, touching and moving objects, opening cupboards, etc.

**Delusions:** the person beMilds untrue things, e.g. insists that someone is trying to hurt or rob them. The person says that their relatives are not who they say they are, or that the house is not their house. This does not apply to when the person appears suspicious; we want to know whether the person is convinced that these things are really happening to them.

**Hallucinations:** the person sees or hears things that do not exist. We do not mean that he is wrongly convinced of something, such as that someone dead is still alive; we want to know whether the person wrongly perceives sounds or visions.

**Severity:** a mild disturbance can be managed easily and has little impact on the person; a severe disturbance is managed with difficulty and/or deeply troubles the person.

**Triggers:** all possible factors that precede the development of behavioral disturbances and are potentially correlated to their onset. ***These do not include the description of the disturbance itself***. For instance, triggers can be arguments with relatives on specific topics, presence of professional caregivers or specific people, need for personal hygiene, heat, cold, etc.

| **Week**  **1** | **Insomnia or fragmented sleep** | **Agitation or anxiety** | **Physical or verbal aggression** | **Purposeless motor behavior** | **Delusions or hallucinations** |
| --- | --- | --- | --- | --- | --- |
| **Monday**  Triggers | Mild: 🞎  Severe: 🞎  Time: | Mild: 🞎  Severe: 🞎  Time: | Mild: 🞎  Severe: 🞎  Time: | Mild: 🞎  Severe: 🞎  Time: | Mild: 🞎  Severe: 🞎  Time: |
|  | _______________  _______________  _______________  _______________ | _______________  _______________  _______________  _______________ | _______________  _______________  _______________  _______________ | _______________  _______________  _______________  _______________ | _______________  _______________  _______________  _______________ |
| **Tuesday**  Triggers | Mild: 🞎  Severe: 🞎  Time: | Mild: 🞎  Severe: 🞎  Time: | Mild: 🞎  Severe: 🞎  Time: | Mild: 🞎  Severe: 🞎  Time: | Mild: 🞎  Severe: 🞎  Time: |
|  | _______________  _______________  _______________  _______________ | _______________  _______________  _______________  _______________ | _______________  _______________  _______________  _______________ | _______________  _______________  _______________  _______________ | _______________  _______________  _______________  _______________ |
| **Wednesday**  Triggers | Mild: 🞎  Severe: 🞎  Time: | Mild: 🞎  Severe: 🞎  Time: | Mild: 🞎  Severe: 🞎  Time: | Mild: 🞎  Severe: 🞎  Time: | Mild: 🞎  Severe: 🞎  Time: |
|  | _______________  _______________  _______________  _______________ | _______________  _______________  _______________  _______________ | _______________  _______________  _______________  _______________ | _______________  _______________  _______________  _______________ | _______________  _______________  _______________  _______________ |
| **Thursday**  Triggers | Mild: 🞎  Severe: 🞎  Time: | Mild: 🞎  Severe: 🞎  Time: | Mild: 🞎  Severe: 🞎  Time: | Mild: 🞎  Severe: 🞎  Time: | Mild: 🞎  Severe: 🞎  Time: |
|  | _______________  _______________  _______________  _______________ | _______________  _______________  _______________  _______________ | _______________  _______________  _______________  _______________ | _______________  _______________  _______________  _______________ | _______________  _______________  _______________  _______________ |
| **Friday**  Triggers | Mild: 🞎  Severe: 🞎  Time: | Mild: 🞎  Severe: 🞎  Time: | Mild: 🞎  Severe: 🞎  Time: | Mild: 🞎  Severe: 🞎  Time: | Mild: 🞎  Severe: 🞎  Time: |
|  | _______________  _______________  _______________  _______________ | _______________  _______________  _______________  _______________ | _______________  _______________  _______________  _______________ | _______________  _______________  _______________  _______________ | _______________  _______________  _______________  _______________ |
| **Saturday**  Triggers | Mild: 🞎  Severe: 🞎  Time: | Mild: 🞎  Severe: 🞎  Time: | Mild: 🞎  Severe: 🞎  Time: | Mild: 🞎  Severe: 🞎  Time: | Mild: 🞎  Severe: 🞎  Time: |
|  | _______________  _______________  _______________  _______________ | _______________  _______________  _______________  _______________ | _______________  _______________  _______________  _______________ | _______________  _______________  _______________  _______________ | _______________  _______________  _______________  _______________ |
| **Sunday**  Triggers | Mild: 🞎  Severe: 🞎  Time: | Mild: 🞎  Severe: 🞎  Time: | Mild: 🞎  Severe: 🞎  Time: | Mild: 🞎  Severe: 🞎  Time: | Mild: 🞎  Severe: 🞎  Time: |
|  | _______________  _______________  _______________  _______________ | _______________  _______________  _______________  _______________ | _______________  _______________  _______________  _______________ | _______________  _______________  _______________  _______________ | _______________  _______________  _______________  _______________ |

| **Week**  **2** | **Insomnia or fragmented sleep** | **Agitation or anxiety** | **Physical or verbal aggression** | **Purposeless motor behavior** | **Delusions or hallucinations** |
| --- | --- | --- | --- | --- | --- |
| **Monday**  Triggers | Mild: 🞎  Severe: 🞎  Time: | Mild: 🞎  Severe: 🞎  Time: | Mild: 🞎  Severe: 🞎  Time: | Mild: 🞎  Severe: 🞎  Time: | Mild: 🞎  Severe: 🞎  Time: |
|  | _______________  _______________  _______________  _______________ | _______________  _______________  _______________  _______________ | _______________  _______________  _______________  _______________ | _______________  _______________  _______________  _______________ | _______________  _______________  _______________  _______________ |
| **Tuesday**  Triggers | Mild: 🞎  Severe: 🞎  Time: | Mild: 🞎  Severe: 🞎  Time: | Mild: 🞎  Severe: 🞎  Time: | Mild: 🞎  Severe: 🞎  Time: | Mild: 🞎  Severe: 🞎  Time: |
|  | _______________  _______________  _______________  _______________ | _______________  _______________  _______________  _______________ | _______________  _______________  _______________  _______________ | _______________  _______________  _______________  _______________ | _______________  _______________  _______________  _______________ |
| **Wednesday**  Triggers | Mild: 🞎  Severe: 🞎  Time: | Mild: 🞎  Severe: 🞎  Time: | Mild: 🞎  Severe: 🞎  Time: | Mild: 🞎  Severe: 🞎  Time: | Mild: 🞎  Severe: 🞎  Time: |
|  | _______________  _______________  _______________  _______________ | _______________  _______________  _______________  _______________ | _______________  _______________  _______________  _______________ | _______________  _______________  _______________  _______________ | _______________  _______________  _______________  _______________ |
| **Thursday**  Triggers | Mild: 🞎  Severe: 🞎  Time: | Mild: 🞎  Severe: 🞎  Time: | Mild: 🞎  Severe: 🞎  Time: | Mild: 🞎  Severe: 🞎  Time: | Mild: 🞎  Severe: 🞎  Time: |
|  | _______________  _______________  _______________  _______________ | _______________  _______________  _______________  _______________ | _______________  _______________  _______________  _______________ | _______________  _______________  _______________  _______________ | _______________  _______________  _______________  _______________ |
| **Friday**  Triggers | Mild: 🞎  Severe: 🞎  Time: | Mild: 🞎  Severe: 🞎  Time: | Mild: 🞎  Severe: 🞎  Time: | Mild: 🞎  Severe: 🞎  Time: | Mild: 🞎  Severe: 🞎  Time: |
|  | _______________  _______________  _______________  _______________ | _______________  _______________  _______________  _______________ | _______________  _______________  _______________  _______________ | _______________  _______________  _______________  _______________ | _______________  _______________  _______________  _______________ |
| **Saturday**  Triggers | Mild: 🞎  Severe: 🞎  Time: | Mild: 🞎  Severe: 🞎  Time: | Mild: 🞎  Severe: 🞎  Time: | Mild: 🞎  Severe: 🞎  Time: | Mild: 🞎  Severe: 🞎  Time: |
|  | _______________  _______________  _______________  _______________ | _______________  _______________  _______________  _______________ | _______________  _______________  _______________  _______________ | _______________  _______________  _______________  _______________ | _______________  _______________  _______________  _______________ |
| **Sunday**  Triggers | Mild: 🞎  Severe: 🞎  Time: | Mild: 🞎  Severe: 🞎  Time: | Mild: 🞎  Severe: 🞎  Time: | Mild: 🞎  Severe: 🞎  Time: | Mild: 🞎  Severe: 🞎  Time: |
|  | _______________  _______________  _______________  _______________ | _______________  _______________  _______________  _______________ | _______________  _______________  _______________  _______________ | _______________  _______________  _______________  _______________ | _______________  _______________  _______________  _______________ |

| **Week**  **3** | **Insomnia or fragmented sleep** | **Agitation or anxiety** | **Physical or verbal aggression** | **Purposeless motor behavior** | **Delusions or hallucinations** |
| --- | --- | --- | --- | --- | --- |
| **Monday**  Triggers | Mild: 🞎  Severe: 🞎  Time: | Mild: 🞎  Severe: 🞎  Time: | Mild: 🞎  Severe: 🞎  Time: | Mild: 🞎  Severe: 🞎  Time: | Mild: 🞎  Severe: 🞎  Time: |
|  | _______________  _______________  _______________  _______________ | _______________  _______________  _______________  _______________ | _______________  _______________  _______________  _______________ | _______________  _______________  _______________  _______________ | _______________  _______________  _______________  _______________ |
| **Tuesday**  Triggers | Mild: 🞎  Severe: 🞎  Time: | Mild: 🞎  Severe: 🞎  Time: | Mild: 🞎  Severe: 🞎  Time: | Mild: 🞎  Severe: 🞎  Time: | Mild: 🞎  Severe: 🞎  Time: |
|  | _______________  _______________  _______________  _______________ | _______________  _______________  _______________  _______________ | _______________  _______________  _______________  _______________ | _______________  _______________  _______________  _______________ | _______________  _______________  _______________  _______________ |
| **Wednesday**  Triggers | Mild: 🞎  Severe: 🞎  Time: | Mild: 🞎  Severe: 🞎  Time: | Mild: 🞎  Severe: 🞎  Time: | Mild: 🞎  Severe: 🞎  Time: | Mild: 🞎  Severe: 🞎  Time: |
|  | _______________  _______________  _______________  _______________ | _______________  _______________  _______________  _______________ | _______________  _______________  _______________  _______________ | _______________  _______________  _______________  _______________ | _______________  _______________  _______________  _______________ |
| **Thursday**  Triggers | Mild: 🞎  Severe: 🞎  Time: | Mild: 🞎  Severe: 🞎  Time: | Mild: 🞎  Severe: 🞎  Time: | Mild: 🞎  Severe: 🞎  Time: | Mild: 🞎  Severe: 🞎  Time: |
|  | _______________  _______________  _______________  _______________ | _______________  _______________  _______________  _______________ | _______________  _______________  _______________  _______________ | _______________  _______________  _______________  _______________ | _______________  _______________  _______________  _______________ |
| **Friday**  Triggers | Mild: 🞎  Severe: 🞎  Time: | Mild: 🞎  Severe: 🞎  Time: | Mild: 🞎  Severe: 🞎  Time: | Mild: 🞎  Severe: 🞎  Time: | Mild: 🞎  Severe: 🞎  Time: |
|  | _______________  _______________  _______________  _______________ | _______________  _______________  _______________  _______________ | _______________  _______________  _______________  _______________ | _______________  _______________  _______________  _______________ | _______________  _______________  _______________  _______________ |
| **Saturday**  Triggers | Mild: 🞎  Severe: 🞎  Time: | Mild: 🞎  Severe: 🞎  Time: | Mild: 🞎  Severe: 🞎  Time: | Mild: 🞎  Severe: 🞎  Time: | Mild: 🞎  Severe: 🞎  Time: |
|  | _______________  _______________  _______________  _______________ | _______________  _______________  _______________  _______________ | _______________  _______________  _______________  _______________ | _______________  _______________  _______________  _______________ | _______________  _______________  _______________  _______________ |
| **Sunday**  Triggers | Mild: 🞎  Severe: 🞎  Time: | Mild: 🞎  Severe: 🞎  Time: | Mild: 🞎  Severe: 🞎  Time: | Mild: 🞎  Severe: 🞎  Time: | Mild: 🞎  Severe: 🞎  Time: |
|  | _______________  _______________  _______________  _______________ | _______________  _______________  _______________  _______________ | _______________  _______________  _______________  _______________ | _______________  _______________  _______________  _______________ | _______________  _______________  _______________  _______________ |

| **Week**  **4** | **Insomnia or fragmented sleep** | **Agitation or anxiety** | **Physical or verbal aggression** | **Purposeless motor behavior** | **Delusions or hallucinations** |
| --- | --- | --- | --- | --- | --- |
| **Monday**  Triggers | Mild: 🞎  Severe: 🞎  Time: | Mild: 🞎  Severe: 🞎  Time: | Mild: 🞎  Severe: 🞎  Time: | Mild: 🞎  Severe: 🞎  Time: | Mild: 🞎  Severe: 🞎  Time: |
|  | _______________  _______________  _______________  _______________ | _______________  _______________  _______________  _______________ | _______________  _______________  _______________  _______________ | _______________  _______________  _______________  _______________ | _______________  _______________  _______________  _______________ |
| **Tuesday**  Triggers | Mild: 🞎  Severe: 🞎  Time: | Mild: 🞎  Severe: 🞎  Time: | Mild: 🞎  Severe: 🞎  Time: | Mild: 🞎  Severe: 🞎  Time: | Mild: 🞎  Severe: 🞎  Time: |
|  | _______________  _______________  _______________  _______________ | _______________  _______________  _______________  _______________ | _______________  _______________  _______________  _______________ | _______________  _______________  _______________  _______________ | _______________  _______________  _______________  _______________ |
| **Wednesday**  Triggers | Mild: 🞎  Severe: 🞎  Time: | Mild: 🞎  Severe: 🞎  Time: | Mild: 🞎  Severe: 🞎  Time: | Mild: 🞎  Severe: 🞎  Time: | Mild: 🞎  Severe: 🞎  Time: |
|  | _______________  _______________  _______________  _______________ | _______________  _______________  _______________  _______________ | _______________  _______________  _______________  _______________ | _______________  _______________  _______________  _______________ | _______________  _______________  _______________  _______________ |
| **Thursday**  Triggers | Mild: 🞎  Severe: 🞎  Time: | Mild: 🞎  Severe: 🞎  Time: | Mild: 🞎  Severe: 🞎  Time: | Mild: 🞎  Severe: 🞎  Time: | Mild: 🞎  Severe: 🞎  Time: |
|  | _______________  _______________  _______________  _______________ | _______________  _______________  _______________  _______________ | _______________  _______________  _______________  _______________ | _______________  _______________  _______________  _______________ | _______________  _______________  _______________  _______________ |
| **Friday**  Triggers | Mild: 🞎  Severe: 🞎  Time: | Mild: 🞎  Severe: 🞎  Time: | Mild: 🞎  Severe: 🞎  Time: | Mild: 🞎  Severe: 🞎  Time: | Mild: 🞎  Severe: 🞎  Time: |
|  | _______________  _______________  _______________  _______________ | _______________  _______________  _______________  _______________ | _______________  _______________  _______________  _______________ | _______________  _______________  _______________  _______________ | _______________  _______________  _______________  _______________ |
| **Saturday**  Triggers | Mild: 🞎  Severe: 🞎  Time: | Mild: 🞎  Severe: 🞎  Time: | Mild: 🞎  Severe: 🞎  Time: | Mild: 🞎  Severe: 🞎  Time: | Mild: 🞎  Severe: 🞎  Time: |
|  | _______________  _______________  _______________  _______________ | _______________  _______________  _______________  _______________ | _______________  _______________  _______________  _______________ | _______________  _______________  _______________  _______________ | _______________  _______________  _______________  _______________ |
| **Sunday**  Triggers | Mild: 🞎  Severe: 🞎  Time: | Mild: 🞎  Severe: 🞎  Time: | Mild: 🞎  Severe: 🞎  Time: | Mild: 🞎  Severe: 🞎  Time: | Mild: 🞎  Severe: 🞎  Time: |
|  | _______________  _______________  _______________  _______________ | _______________  _______________  _______________  _______________ | _______________  _______________  _______________  _______________ | _______________  _______________  _______________  _______________ | _______________  _______________  _______________  _______________ |

| **Week**  **5** | **Insomnia or fragmented sleep** | **Agitation or anxiety** | **Physical or verbal aggression** | **Purposeless motor behavior** | **Delusions or hallucinations** |
| --- | --- | --- | --- | --- | --- |
| **Monday**  Triggers | Mild: 🞎  Severe: 🞎  Time: | Mild: 🞎  Severe: 🞎  Time: | Mild: 🞎  Severe: 🞎  Time: | Mild: 🞎  Severe: 🞎  Time: | Mild: 🞎  Severe: 🞎  Time: |
|  | _______________  _______________  _______________  _______________ | _______________  _______________  _______________  _______________ | _______________  _______________  _______________  _______________ | _______________  _______________  _______________  _______________ | _______________  _______________  _______________  _______________ |
| **Tuesday**  Triggers | Mild: 🞎  Severe: 🞎  Time: | Mild: 🞎  Severe: 🞎  Time: | Mild: 🞎  Severe: 🞎  Time: | Mild: 🞎  Severe: 🞎  Time: | Mild: 🞎  Severe: 🞎  Time: |
|  | _______________  _______________  _______________  _______________ | _______________  _______________  _______________  _______________ | _______________  _______________  _______________  _______________ | _______________  _______________  _______________  _______________ | _______________  _______________  _______________  _______________ |
| **Wednesday**  Triggers | Mild: 🞎  Severe: 🞎  Time: | Mild: 🞎  Severe: 🞎  Time: | Mild: 🞎  Severe: 🞎  Time: | Mild: 🞎  Severe: 🞎  Time: | Mild: 🞎  Severe: 🞎  Time: |
|  | _______________  _______________  _______________  _______________ | _______________  _______________  _______________  _______________ | _______________  _______________  _______________  _______________ | _______________  _______________  _______________  _______________ | _______________  _______________  _______________  _______________ |
| **Thursday**  Triggers | Mild: 🞎  Severe: 🞎  Time: | Mild: 🞎  Severe: 🞎  Time: | Mild: 🞎  Severe: 🞎  Time: | Mild: 🞎  Severe: 🞎  Time: | Mild: 🞎  Severe: 🞎  Time: |
|  | _______________  _______________  _______________  _______________ | _______________  _______________  _______________  _______________ | _______________  _______________  _______________  _______________ | _______________  _______________  _______________  _______________ | _______________  _______________  _______________  _______________ |
| **Friday**  Triggers | Mild: 🞎  Severe: 🞎  Time: | Mild: 🞎  Severe: 🞎  Time: | Mild: 🞎  Severe: 🞎  Time: | Mild: 🞎  Severe: 🞎  Time: | Mild: 🞎  Severe: 🞎  Time: |
|  | _______________  _______________  _______________  _______________ | _______________  _______________  _______________  _______________ | _______________  _______________  _______________  _______________ | _______________  _______________  _______________  _______________ | _______________  _______________  _______________  _______________ |
| **Saturday**  Triggers | Mild: 🞎  Severe: 🞎  Time: | Mild: 🞎  Severe: 🞎  Time: | Mild: 🞎  Severe: 🞎  Time: | Mild: 🞎  Severe: 🞎  Time: | Mild: 🞎  Severe: 🞎  Time: |
|  | _______________  _______________  _______________  _______________ | _______________  _______________  _______________  _______________ | _______________  _______________  _______________  _______________ | _______________  _______________  _______________  _______________ | _______________  _______________  _______________  _______________ |
| **Sunday**  Triggers | Mild: 🞎  Severe: 🞎  Time: | Mild: 🞎  Severe: 🞎  Time: | Mild: 🞎  Severe: 🞎  Time: | Mild: 🞎  Severe: 🞎  Time: | Mild: 🞎  Severe: 🞎  Time: |
|  | _______________  _______________  _______________  _______________ | _______________  _______________  _______________  _______________ | _______________  _______________  _______________  _______________ | _______________  _______________  _______________  _______________ | _______________  _______________  _______________  _______________ |

| **Week**  **6** | **Insomnia or fragmented sleep** | **Agitation or anxiety** | **Physical or verbal aggression** | **Purposeless motor behavior** | **Delusions or hallucinations** |
| --- | --- | --- | --- | --- | --- |
| **Monday**  Triggers | Mild: 🞎  Severe: 🞎  Time: | Mild: 🞎  Severe: 🞎  Time: | Mild: 🞎  Severe: 🞎  Time: | Mild: 🞎  Severe: 🞎  Time: | Mild: 🞎  Severe: 🞎  Time: |
|  | _______________  _______________  _______________  _______________ | _______________  _______________  _______________  _______________ | _______________  _______________  _______________  _______________ | _______________  _______________  _______________  _______________ | _______________  _______________  _______________  _______________ |
| **Tuesday**  Triggers | Mild: 🞎  Severe: 🞎  Time: | Mild: 🞎  Severe: 🞎  Time: | Mild: 🞎  Severe: 🞎  Time: | Mild: 🞎  Severe: 🞎  Time: | Mild: 🞎  Severe: 🞎  Time: |
|  | _______________  _______________  _______________  _______________ | _______________  _______________  _______________  _______________ | _______________  _______________  _______________  _______________ | _______________  _______________  _______________  _______________ | _______________  _______________  _______________  _______________ |
| **Wednesday**  Triggers | Mild: 🞎  Severe: 🞎  Time: | Mild: 🞎  Severe: 🞎  Time: | Mild: 🞎  Severe: 🞎  Time: | Mild: 🞎  Severe: 🞎  Time: | Mild: 🞎  Severe: 🞎  Time: |
|  | _______________  _______________  _______________  _______________ | _______________  _______________  _______________  _______________ | _______________  _______________  _______________  _______________ | _______________  _______________  _______________  _______________ | _______________  _______________  _______________  _______________ |
| **Thursday**  Triggers | Mild: 🞎  Severe: 🞎  Time: | Mild: 🞎  Severe: 🞎  Time: | Mild: 🞎  Severe: 🞎  Time: | Mild: 🞎  Severe: 🞎  Time: | Mild: 🞎  Severe: 🞎  Time: |
|  | _______________  _______________  _______________  _______________ | _______________  _______________  _______________  _______________ | _______________  _______________  _______________  _______________ | _______________  _______________  _______________  _______________ | _______________  _______________  _______________  _______________ |
| **Friday**  Triggers | Mild: 🞎  Severe: 🞎  Time: | Mild: 🞎  Severe: 🞎  Time: | Mild: 🞎  Severe: 🞎  Time: | Mild: 🞎  Severe: 🞎  Time: | Mild: 🞎  Severe: 🞎  Time: |
|  | _______________  _______________  _______________  _______________ | _______________  _______________  _______________  _______________ | _______________  _______________  _______________  _______________ | _______________  _______________  _______________  _______________ | _______________  _______________  _______________  _______________ |
| **Saturday**  Triggers | Mild: 🞎  Severe: 🞎  Time: | Mild: 🞎  Severe: 🞎  Time: | Mild: 🞎  Severe: 🞎  Time: | Mild: 🞎  Severe: 🞎  Time: | Mild: 🞎  Severe: 🞎  Time: |
|  | _______________  _______________  _______________  _______________ | _______________  _______________  _______________  _______________ | _______________  _______________  _______________  _______________ | _______________  _______________  _______________  _______________ | _______________  _______________  _______________  _______________ |
| **Sunday**  Triggers | Mild: 🞎  Severe: 🞎  Time: | Mild: 🞎  Severe: 🞎  Time: | Mild: 🞎  Severe: 🞎  Time: | Mild: 🞎  Severe: 🞎  Time: | Mild: 🞎  Severe: 🞎  Time: |
|  | _______________  _______________  _______________  _______________ | _______________  _______________  _______________  _______________ | _______________  _______________  _______________  _______________ | _______________  _______________  _______________  _______________ | _______________  _______________  _______________  _______________ |

| **Week**  **7** | **Insomnia or fragmented sleep** | **Agitation or anxiety** | **Physical or verbal aggression** | **Purposeless motor behavior** | **Delusions or hallucinations** |
| --- | --- | --- | --- | --- | --- |
| **Monday**  Triggers | Mild: 🞎  Severe: 🞎  Time: | Mild: 🞎  Severe: 🞎  Time: | Mild: 🞎  Severe: 🞎  Time: | Mild: 🞎  Severe: 🞎  Time: | Mild: 🞎  Severe: 🞎  Time: |
|  | _______________  _______________  _______________  _______________ | _______________  _______________  _______________  _______________ | _______________  _______________  _______________  _______________ | _______________  _______________  _______________  _______________ | _______________  _______________  _______________  _______________ |
| **Tuesday**  Triggers | Mild: 🞎  Severe: 🞎  Time: | Mild: 🞎  Severe: 🞎  Time: | Mild: 🞎  Severe: 🞎  Time: | Mild: 🞎  Severe: 🞎  Time: | Mild: 🞎  Severe: 🞎  Time: |
|  | _______________  _______________  _______________  _______________ | _______________  _______________  _______________  _______________ | _______________  _______________  _______________  _______________ | _______________  _______________  _______________  _______________ | _______________  _______________  _______________  _______________ |
| **Wednesday**  Triggers | Mild: 🞎  Severe: 🞎  Time: | Mild: 🞎  Severe: 🞎  Time: | Mild: 🞎  Severe: 🞎  Time: | Mild: 🞎  Severe: 🞎  Time: | Mild: 🞎  Severe: 🞎  Time: |
|  | _______________  _______________  _______________  _______________ | _______________  _______________  _______________  _______________ | _______________  _______________  _______________  _______________ | _______________  _______________  _______________  _______________ | _______________  _______________  _______________  _______________ |
| **Thursday**  Triggers | Mild: 🞎  Severe: 🞎  Time: | Mild: 🞎  Severe: 🞎  Time: | Mild: 🞎  Severe: 🞎  Time: | Mild: 🞎  Severe: 🞎  Time: | Mild: 🞎  Severe: 🞎  Time: |
|  | _______________  _______________  _______________  _______________ | _______________  _______________  _______________  _______________ | _______________  _______________  _______________  _______________ | _______________  _______________  _______________  _______________ | _______________  _______________  _______________  _______________ |
| **Friday**  Triggers | Mild: 🞎  Severe: 🞎  Time: | Mild: 🞎  Severe: 🞎  Time: | Mild: 🞎  Severe: 🞎  Time: | Mild: 🞎  Severe: 🞎  Time: | Mild: 🞎  Severe: 🞎  Time: |
|  | _______________  _______________  _______________  _______________ | _______________  _______________  _______________  _______________ | _______________  _______________  _______________  _______________ | _______________  _______________  _______________  _______________ | _______________  _______________  _______________  _______________ |
| **Saturday**  Triggers | Mild: 🞎  Severe: 🞎  Time: | Mild: 🞎  Severe: 🞎  Time: | Mild: 🞎  Severe: 🞎  Time: | Mild: 🞎  Severe: 🞎  Time: | Mild: 🞎  Severe: 🞎  Time: |
|  | _______________  _______________  _______________  _______________ | _______________  _______________  _______________  _______________ | _______________  _______________  _______________  _______________ | _______________  _______________  _______________  _______________ | _______________  _______________  _______________  _______________ |
| **Sunday**  Triggers | Mild: 🞎  Severe: 🞎  Time: | Mild: 🞎  Severe: 🞎  Time: | Mild: 🞎  Severe: 🞎  Time: | Mild: 🞎  Severe: 🞎  Time: | Mild: 🞎  Severe: 🞎  Time: |
|  | _______________  _______________  _______________  _______________ | _______________  _______________  _______________  _______________ | _______________  _______________  _______________  _______________ | _______________  _______________  _______________  _______________ | _______________  _______________  _______________  _______________ |

| **Week**  **8** | **Insomnia or fragmented sleep** | **Agitation or anxiety** | **Physical or verbal aggression** | **Purposeless motor behavior** | **Delusions or hallucinations** |
| --- | --- | --- | --- | --- | --- |
| **Monday**  Triggers | Mild: 🞎  Severe: 🞎  Time: | Mild: 🞎  Severe: 🞎  Time: | Mild: 🞎  Severe: 🞎  Time: | Mild: 🞎  Severe: 🞎  Time: | Mild: 🞎  Severe: 🞎  Time: |
|  | _______________  _______________  _______________  _______________ | _______________  _______________  _______________  _______________ | _______________  _______________  _______________  _______________ | _______________  _______________  _______________  _______________ | _______________  _______________  _______________  _______________ |
| **Tuesday**  Triggers | Mild: 🞎  Severe: 🞎  Time: | Mild: 🞎  Severe: 🞎  Time: | Mild: 🞎  Severe: 🞎  Time: | Mild: 🞎  Severe: 🞎  Time: | Mild: 🞎  Severe: 🞎  Time: |
|  | _______________  _______________  _______________  _______________ | _______________  _______________  _______________  _______________ | _______________  _______________  _______________  _______________ | _______________  _______________  _______________  _______________ | _______________  _______________  _______________  _______________ |
| **Wednesday**  Triggers | Mild: 🞎  Severe: 🞎  Time: | Mild: 🞎  Severe: 🞎  Time: | Mild: 🞎  Severe: 🞎  Time: | Mild: 🞎  Severe: 🞎  Time: | Mild: 🞎  Severe: 🞎  Time: |
|  | _______________  _______________  _______________  _______________ | _______________  _______________  _______________  _______________ | _______________  _______________  _______________  _______________ | _______________  _______________  _______________  _______________ | _______________  _______________  _______________  _______________ |
| **Thursday**  Triggers | Mild: 🞎  Severe: 🞎  Time: | Mild: 🞎  Severe: 🞎  Time: | Mild: 🞎  Severe: 🞎  Time: | Mild: 🞎  Severe: 🞎  Time: | Mild: 🞎  Severe: 🞎  Time: |
|  | _______________  _______________  _______________  _______________ | _______________  _______________  _______________  _______________ | _______________  _______________  _______________  _______________ | _______________  _______________  _______________  _______________ | _______________  _______________  _______________  _______________ |
| **Friday**  Triggers | Mild: 🞎  Severe: 🞎  Time: | Mild: 🞎  Severe: 🞎  Time: | Mild: 🞎  Severe: 🞎  Time: | Mild: 🞎  Severe: 🞎  Time: | Mild: 🞎  Severe: 🞎  Time: |
|  | _______________  _______________  _______________  _______________ | _______________  _______________  _______________  _______________ | _______________  _______________  _______________  _______________ | _______________  _______________  _______________  _______________ | _______________  _______________  _______________  _______________ |
| **Saturday**  Triggers | Mild: 🞎  Severe: 🞎  Time: | Mild: 🞎  Severe: 🞎  Time: | Mild: 🞎  Severe: 🞎  Time: | Mild: 🞎  Severe: 🞎  Time: | Mild: 🞎  Severe: 🞎  Time: |
|  | _______________  _______________  _______________  _______________ | _______________  _______________  _______________  _______________ | _______________  _______________  _______________  _______________ | _______________  _______________  _______________  _______________ | _______________  _______________  _______________  _______________ |
| **Sunday**  Triggers | Mild: 🞎  Severe: 🞎  Time: | Mild: 🞎  Severe: 🞎  Time: | Mild: 🞎  Severe: 🞎  Time: | Mild: 🞎  Severe: 🞎  Time: | Mild: 🞎  Severe: 🞎  Time: |
|  | _______________  _______________  _______________  _______________ | _______________  _______________  _______________  _______________ | _______________  _______________  _______________  _______________ | _______________  _______________  _______________  _______________ | _______________  _______________  _______________  _______________ |

| **Week**  **9** | **Insomnia or fragmented sleep** | **Agitation or anxiety** | **Physical or verbal aggression** | **Purposeless motor behavior** | **Delusions or hallucinations** |
| --- | --- | --- | --- | --- | --- |
| **Monday**  Triggers | Mild: 🞎  Severe: 🞎  Time: | Mild: 🞎  Severe: 🞎  Time: | Mild: 🞎  Severe: 🞎  Time: | Mild: 🞎  Severe: 🞎  Time: | Mild: 🞎  Severe: 🞎  Time: |
|  | _______________  _______________  _______________  _______________ | _______________  _______________  _______________  _______________ | _______________  _______________  _______________  _______________ | _______________  _______________  _______________  _______________ | _______________  _______________  _______________  _______________ |
| **Tuesday**  Triggers | Mild: 🞎  Severe: 🞎  Time: | Mild: 🞎  Severe: 🞎  Time: | Mild: 🞎  Severe: 🞎  Time: | Mild: 🞎  Severe: 🞎  Time: | Mild: 🞎  Severe: 🞎  Time: |
|  | _______________  _______________  _______________  _______________ | _______________  _______________  _______________  _______________ | _______________  _______________  _______________  _______________ | _______________  _______________  _______________  _______________ | _______________  _______________  _______________  _______________ |
| **Wednesday**  Triggers | Mild: 🞎  Severe: 🞎  Time: | Mild: 🞎  Severe: 🞎  Time: | Mild: 🞎  Severe: 🞎  Time: | Mild: 🞎  Severe: 🞎  Time: | Mild: 🞎  Severe: 🞎  Time: |
|  | _______________  _______________  _______________  _______________ | _______________  _______________  _______________  _______________ | _______________  _______________  _______________  _______________ | _______________  _______________  _______________  _______________ | _______________  _______________  _______________  _______________ |
| **Thursday**  Triggers | Mild: 🞎  Severe: 🞎  Time: | Mild: 🞎  Severe: 🞎  Time: | Mild: 🞎  Severe: 🞎  Time: | Mild: 🞎  Severe: 🞎  Time: | Mild: 🞎  Severe: 🞎  Time: |
|  | _______________  _______________  _______________  _______________ | _______________  _______________  _______________  _______________ | _______________  _______________  _______________  _______________ | _______________  _______________  _______________  _______________ | _______________  _______________  _______________  _______________ |
| **Friday**  Triggers | Mild: 🞎  Severe: 🞎  Time: | Mild: 🞎  Severe: 🞎  Time: | Mild: 🞎  Severe: 🞎  Time: | Mild: 🞎  Severe: 🞎  Time: | Mild: 🞎  Severe: 🞎  Time: |
|  | _______________  _______________  _______________  _______________ | _______________  _______________  _______________  _______________ | _______________  _______________  _______________  _______________ | _______________  _______________  _______________  _______________ | _______________  _______________  _______________  _______________ |
| **Saturday**  Triggers | Mild: 🞎  Severe: 🞎  Time: | Mild: 🞎  Severe: 🞎  Time: | Mild: 🞎  Severe: 🞎  Time: | Mild: 🞎  Severe: 🞎  Time: | Mild: 🞎  Severe: 🞎  Time: |
|  | _______________  _______________  _______________  _______________ | _______________  _______________  _______________  _______________ | _______________  _______________  _______________  _______________ | _______________  _______________  _______________  _______________ | _______________  _______________  _______________  _______________ |
| **Sunday**  Triggers | Mild: 🞎  Severe: 🞎  Time: | Mild: 🞎  Severe: 🞎  Time: | Mild: 🞎  Severe: 🞎  Time: | Mild: 🞎  Severe: 🞎  Time: | Mild: 🞎  Severe: 🞎  Time: |
|  | _______________  _______________  _______________  _______________ | _______________  _______________  _______________  _______________ | _______________  _______________  _______________  _______________ | _______________  _______________  _______________  _______________ | _______________  _______________  _______________  _______________ |

| **Week**  **10** | **Insomnia or fragmented sleep** | **Agitation or anxiety** | **Physical or verbal aggression** | **Purposeless motor behavior** | **Delusions or hallucinations** |
| --- | --- | --- | --- | --- | --- |
| **Monday**  Triggers | Mild: 🞎  Severe: 🞎  Time: | Mild: 🞎  Severe: 🞎  Time: | Mild: 🞎  Severe: 🞎  Time: | Mild: 🞎  Severe: 🞎  Time: | Mild: 🞎  Severe: 🞎  Time: |
|  | _______________  _______________  _______________  _______________ | _______________  _______________  _______________  _______________ | _______________  _______________  _______________  _______________ | _______________  _______________  _______________  _______________ | _______________  _______________  _______________  _______________ |
| **Tuesday**  Triggers | Mild: 🞎  Severe: 🞎  Time: | Mild: 🞎  Severe: 🞎  Time: | Mild: 🞎  Severe: 🞎  Time: | Mild: 🞎  Severe: 🞎  Time: | Mild: 🞎  Severe: 🞎  Time: |
|  | _______________  _______________  _______________  _______________ | _______________  _______________  _______________  _______________ | _______________  _______________  _______________  _______________ | _______________  _______________  _______________  _______________ | _______________  _______________  _______________  _______________ |
| **Wednesday**  Triggers | Mild: 🞎  Severe: 🞎  Time: | Mild: 🞎  Severe: 🞎  Time: | Mild: 🞎  Severe: 🞎  Time: | Mild: 🞎  Severe: 🞎  Time: | Mild: 🞎  Severe: 🞎  Time: |
|  | _______________  _______________  _______________  _______________ | _______________  _______________  _______________  _______________ | _______________  _______________  _______________  _______________ | _______________  _______________  _______________  _______________ | _______________  _______________  _______________  _______________ |
| **Thursday**  Triggers | Mild: 🞎  Severe: 🞎  Time: | Mild: 🞎  Severe: 🞎  Time: | Mild: 🞎  Severe: 🞎  Time: | Mild: 🞎  Severe: 🞎  Time: | Mild: 🞎  Severe: 🞎  Time: |
|  | _______________  _______________  _______________  _______________ | _______________  _______________  _______________  _______________ | _______________  _______________  _______________  _______________ | _______________  _______________  _______________  _______________ | _______________  _______________  _______________  _______________ |
| **Friday**  Triggers | Mild: 🞎  Severe: 🞎  Time: | Mild: 🞎  Severe: 🞎  Time: | Mild: 🞎  Severe: 🞎  Time: | Mild: 🞎  Severe: 🞎  Time: | Mild: 🞎  Severe: 🞎  Time: |
|  | _______________  _______________  _______________  _______________ | _______________  _______________  _______________  _______________ | _______________  _______________  _______________  _______________ | _______________  _______________  _______________  _______________ | _______________  _______________  _______________  _______________ |
| **Saturday**  Triggers | Mild: 🞎  Severe: 🞎  Time: | Mild: 🞎  Severe: 🞎  Time: | Mild: 🞎  Severe: 🞎  Time: | Mild: 🞎  Severe: 🞎  Time: | Mild: 🞎  Severe: 🞎  Time: |
|  | _______________  _______________  _______________  _______________ | _______________  _______________  _______________  _______________ | _______________  _______________  _______________  _______________ | _______________  _______________  _______________  _______________ | _______________  _______________  _______________  _______________ |
| **Sunday**  Triggers | Mild: 🞎  Severe: 🞎  Time: | Mild: 🞎  Severe: 🞎  Time: | Mild: 🞎  Severe: 🞎  Time: | Mild: 🞎  Severe: 🞎  Time: | Mild: 🞎  Severe: 🞎  Time: |
|  | _______________  _______________  _______________  _______________ | _______________  _______________  _______________  _______________ | _______________  _______________  _______________  _______________ | _______________  _______________  _______________  _______________ | _______________  _______________  _______________  _______________ |

| **Week**  **11** | **Insomnia or fragmented sleep** | **Agitation or anxiety** | **Physical or verbal aggression** | **Purposeless motor behavior** | **Delusions or hallucinations** |
| --- | --- | --- | --- | --- | --- |
| **Monday**  Triggers | Mild: 🞎  Severe: 🞎  Time: | Mild: 🞎  Severe: 🞎  Time: | Mild: 🞎  Severe: 🞎  Time: | Mild: 🞎  Severe: 🞎  Time: | Mild: 🞎  Severe: 🞎  Time: |
|  | _______________  _______________  _______________  _______________ | _______________  _______________  _______________  _______________ | _______________  _______________  _______________  _______________ | _______________  _______________  _______________  _______________ | _______________  _______________  _______________  _______________ |
| **Tuesday**  Triggers | Mild: 🞎  Severe: 🞎  Time: | Mild: 🞎  Severe: 🞎  Time: | Mild: 🞎  Severe: 🞎  Time: | Mild: 🞎  Severe: 🞎  Time: | Mild: 🞎  Severe: 🞎  Time: |
|  | _______________  _______________  _______________  _______________ | _______________  _______________  _______________  _______________ | _______________  _______________  _______________  _______________ | _______________  _______________  _______________  _______________ | _______________  _______________  _______________  _______________ |
| **Wednesday**  Triggers | Mild: 🞎  Severe: 🞎  Time: | Mild: 🞎  Severe: 🞎  Time: | Mild: 🞎  Severe: 🞎  Time: | Mild: 🞎  Severe: 🞎  Time: | Mild: 🞎  Severe: 🞎  Time: |
|  | _______________  _______________  _______________  _______________ | _______________  _______________  _______________  _______________ | _______________  _______________  _______________  _______________ | _______________  _______________  _______________  _______________ | _______________  _______________  _______________  _______________ |
| **Thursday**  Triggers | Mild: 🞎  Severe: 🞎  Time: | Mild: 🞎  Severe: 🞎  Time: | Mild: 🞎  Severe: 🞎  Time: | Mild: 🞎  Severe: 🞎  Time: | Mild: 🞎  Severe: 🞎  Time: |
|  | _______________  _______________  _______________  _______________ | _______________  _______________  _______________  _______________ | _______________  _______________  _______________  _______________ | _______________  _______________  _______________  _______________ | _______________  _______________  _______________  _______________ |
| **Friday**  Triggers | Mild: 🞎  Severe: 🞎  Time: | Mild: 🞎  Severe: 🞎  Time: | Mild: 🞎  Severe: 🞎  Time: | Mild: 🞎  Severe: 🞎  Time: | Mild: 🞎  Severe: 🞎  Time: |
|  | _______________  _______________  _______________  _______________ | _______________  _______________  _______________  _______________ | _______________  _______________  _______________  _______________ | _______________  _______________  _______________  _______________ | _______________  _______________  _______________  _______________ |
| **Saturday**  Triggers | Mild: 🞎  Severe: 🞎  Time: | Mild: 🞎  Severe: 🞎  Time: | Mild: 🞎  Severe: 🞎  Time: | Mild: 🞎  Severe: 🞎  Time: | Mild: 🞎  Severe: 🞎  Time: |
|  | _______________  _______________  _______________  _______________ | _______________  _______________  _______________  _______________ | _______________  _______________  _______________  _______________ | _______________  _______________  _______________  _______________ | _______________  _______________  _______________  _______________ |
| **Sunday**  Triggers | Mild: 🞎  Severe: 🞎  Time: | Mild: 🞎  Severe: 🞎  Time: | Mild: 🞎  Severe: 🞎  Time: | Mild: 🞎  Severe: 🞎  Time: | Mild: 🞎  Severe: 🞎  Time: |
|  | _______________  _______________  _______________  _______________ | _______________  _______________  _______________  _______________ | _______________  _______________  _______________  _______________ | _______________  _______________  _______________  _______________ | _______________  _______________  _______________  _______________ |

| **Week**  **12** | **Insomnia or fragmented sleep** | **Agitation or anxiety** | **Physical or verbal aggression** | **Purposeless motor behavior** | **Delusions or hallucinations** |
| --- | --- | --- | --- | --- | --- |
| **Monday**  Triggers | Mild: 🞎  Severe: 🞎  Time: | Mild: 🞎  Severe: 🞎  Time: | Mild: 🞎  Severe: 🞎  Time: | Mild: 🞎  Severe: 🞎  Time: | Mild: 🞎  Severe: 🞎  Time: |
|  | _______________  _______________  _______________  _______________ | _______________  _______________  _______________  _______________ | _______________  _______________  _______________  _______________ | _______________  _______________  _______________  _______________ | _______________  _______________  _______________  _______________ |
| **Tuesday**  Triggers | Mild: 🞎  Severe: 🞎  Time: | Mild: 🞎  Severe: 🞎  Time: | Mild: 🞎  Severe: 🞎  Time: | Mild: 🞎  Severe: 🞎  Time: | Mild: 🞎  Severe: 🞎  Time: |
|  | _______________  _______________  _______________  _______________ | _______________  _______________  _______________  _______________ | _______________  _______________  _______________  _______________ | _______________  _______________  _______________  _______________ | _______________  _______________  _______________  _______________ |
| **Wednesday**  Triggers | Mild: 🞎  Severe: 🞎  Time: | Mild: 🞎  Severe: 🞎  Time: | Mild: 🞎  Severe: 🞎  Time: | Mild: 🞎  Severe: 🞎  Time: | Mild: 🞎  Severe: 🞎  Time: |
|  | _______________  _______________  _______________  _______________ | _______________  _______________  _______________  _______________ | _______________  _______________  _______________  _______________ | _______________  _______________  _______________  _______________ | _______________  _______________  _______________  _______________ |
| **Thursday**  Triggers | Mild: 🞎  Severe: 🞎  Time: | Mild: 🞎  Severe: 🞎  Time: | Mild: 🞎  Severe: 🞎  Time: | Mild: 🞎  Severe: 🞎  Time: | Mild: 🞎  Severe: 🞎  Time: |
|  | _______________  _______________  _______________  _______________ | _______________  _______________  _______________  _______________ | _______________  _______________  _______________  _______________ | _______________  _______________  _______________  _______________ | _______________  _______________  _______________  _______________ |
| **Friday**  Triggers | Mild: 🞎  Severe: 🞎  Time: | Mild: 🞎  Severe: 🞎  Time: | Mild: 🞎  Severe: 🞎  Time: | Mild: 🞎  Severe: 🞎  Time: | Mild: 🞎  Severe: 🞎  Time: |
|  | _______________  _______________  _______________  _______________ | _______________  _______________  _______________  _______________ | _______________  _______________  _______________  _______________ | _______________  _______________  _______________  _______________ | _______________  _______________  _______________  _______________ |
| **Saturday**  Triggers | Mild: 🞎  Severe: 🞎  Time: | Mild: 🞎  Severe: 🞎  Time: | Mild: 🞎  Severe: 🞎  Time: | Mild: 🞎  Severe: 🞎  Time: | Mild: 🞎  Severe: 🞎  Time: |
|  | _______________  _______________  _______________  _______________ | _______________  _______________  _______________  _______________ | _______________  _______________  _______________  _______________ | _______________  _______________  _______________  _______________ | _______________  _______________  _______________  _______________ |
| **Sunday**  Triggers | Mild: 🞎  Severe: 🞎  Time: | Mild: 🞎  Severe: 🞎  Time: | Mild: 🞎  Severe: 🞎  Time: | Mild: 🞎  Severe: 🞎  Time: | Mild: 🞎  Severe: 🞎  Time: |
|  | _______________  _______________  _______________  _______________ | _______________  _______________  _______________  _______________ | _______________  _______________  _______________  _______________ | _______________  _______________  _______________  _______________ | _______________  _______________  _______________  _______________ |
