# Supplementary material for: BPSDiary study protocol: a multi-center randomized controlled trial to compare the efficacy of a BPSD diary vs. standard care in reducing caregiver's burden
Source: Front Dement. 2023 Dec 18;2:1301280. doi: 10.3389/frdem.2023.1301280 (PMC11285609; doi:10.3389/frdem.2023.1301280)
Supplement: Supplementary file 4 [file Data_Sheet_2.docx]

**Diario clinico a cura del caregiver**

Paziente: Sig./Sig.ra _________________________________ nato/a il ___/___/____

Caregiver: Sig./Sig.ra _________________________________ nato/a il ___/___/____

grado di parentela/legame con il/la paziente: _________________________________

Data di inizio compilazione diario: ___/___/____

**Introduzione**

**Scopo del diario:**

Il diario è uno strumento per la rilevazione dei disturbi comportamentali e psicologici associati alla demenza. Si tratta di un modo per rendere più oggettiva la comunicazione con il medico che segue il paziente, oltre che per individuare strategie più efficaci per la gestione di tali problematiche.

Compili il diario nel modo più completo possibile, registrando giornalmente l’eventuale presenza dei disturbi, il loro orario, gravità ed eventuali fattori scatenanti. Per stabilire la categoria di disturbo presente, faccia riferimento alle definizioni qui sotto. Se ha dubbi, consulti lo specialista che le ha fornito il diario.

**Definizioni:**

**Insonnia o sonno frammentato:** il paziente non riesce a dormire, si sveglia spesso di notte, si alza troppo presto al mattino o si appisola spesso durante il giorno. Oppure sta alzato, vaga per la casa durante la notte, si veste e si sveste, disturba il sonno dei familiari. Non è da considerare se il paziente si alza due o tre volte per notte per andare in bagno e poi torna a letto e si addormenta immediatamente.

**Agitazione:** il paziente è intollerante, smanioso, irritabile, capriccioso, è impaziente, non tollera ritardi o attese ad appuntamenti (per esempio visite mediche).

**Ansia:** il paziente è molto nervoso, allarmato, spaventato senza veri motivi, sembra molto teso o agitato, è impaurito dal rimanere lontano dal caregiver.

**Aggressività fisica e verbale:** il paziente si ribella verbalmente e/o fisicamente all’aiuto e all’assistenza, oppure è insulta o colpisce familiari o altre persone senza una causa proporzionata.

**Attività motoria aberrante:** il paziente compie attività ripetitive o prive di scopo, come camminare intorno a casa (wandering, vagabondaggio), sbottonarsi, slacciarsi le scarpe, toccare e spostare gli oggetti, aprire gli armadi, ecc.

**Deliri:** il paziente crede cose che non sono vere, per esempio, insiste sul fatto che qualcuno sta cercando di fargli del male o di rubargli qualcosa. Dice che i componenti della famiglia non sono chi dicono di essere, o che la casa non è la sua. Non ci si riferisce a semplice sospettosità; siamo interessati a sapere se il paziente sia convinto che queste cose gli stiano realmente accadendo.

**Allucinazioni:** il paziente vede o sente cose che non esistono. Con questo non intendiamo solamente convinzioni sbagliate, come per esempio affermare che una persona morta sia ancora viva; piuttosto vogliamo sapere se il paziente presenta la percezione non normale di suoni o di visioni.

**Grado:** per disturbo comportamentale lieve si intende un disturbo che può essere gestito facilmente e di poco impatto per il paziente; per disturbo comportamentale grave si intende un disturbo di difficile gestione e/o che reca grande disturbo al paziente.

**Fattori scatenanti:** tutti i possibili fattori che precedono lo sviluppo dei disturbi comportamentali e che possono essere potenzialmente correlabili alla loro insorgenza. Tali fattori non includono la descrizione del disturbo. Per esempio, fattori scatenanti possono essere discussioni con i familiari su specifici argomenti, presenza di badanti o di altre persone specifiche, necessità di igiene personale, caldo, buio, ecc

| **Settimana**  **1** | **Insonnia o sonno**  **frammentato** | **Agitazione o ansia** | **Aggressività fisica o verbale** | **Attività motoria aberrante** | **Deliri o allucinazioni** |
| --- | --- | --- | --- | --- | --- |
| **Lunedì**  Fattori scatenanti | Lieve: 🞎  Grave: 🞎  Orario: | Lieve: 🞎  Grave: 🞎  Orario: | Lieve: 🞎  Grave: 🞎  Orario: | Lieve: 🞎  Grave: 🞎  Orario: | Lieve: 🞎  Grave: 🞎  Orario: |
|  | _______________  _______________  _______________  _______________ | _______________  _______________  _______________  _______________ | _______________  _______________  _______________  _______________ | _______________  _______________  _______________  _______________ | _______________  _______________  _______________  _______________ |
| **Martedì**  Fattori scatenanti | Lieve: 🞎  Grave: 🞎  Orario: | Lieve: 🞎  Grave: 🞎  Orario: | Lieve: 🞎  Grave: 🞎  Orario: | Lieve: 🞎  Grave: 🞎  Orario: | Lieve: 🞎  Grave: 🞎  Orario: |
|  | _______________  _______________  _______________  _______________ | _______________  _______________  _______________  _______________ | _______________  _______________  _______________  _______________ | _______________  _______________  _______________  _______________ | _______________  _______________  _______________  _______________ |
| **Mercoledì**  Fattori scatenanti | Lieve: 🞎  Grave: 🞎  Orario: | Lieve: 🞎  Grave: 🞎  Orario: | Lieve: 🞎  Grave: 🞎  Orario: | Lieve: 🞎  Grave: 🞎  Orario: | Lieve: 🞎  Grave: 🞎  Orario: |
|  | _______________  _______________  _______________  _______________ | _______________  _______________  _______________  _______________ | _______________  _______________  _______________  _______________ | _______________  _______________  _______________  _______________ | _______________  _______________  _______________  _______________ |
| **Giovedì**  Fattori scatenanti | Lieve: 🞎  Grave: 🞎  Orario: | Lieve: 🞎  Grave: 🞎  Orario: | Lieve: 🞎  Grave: 🞎  Orario: | Lieve: 🞎  Grave: 🞎  Orario: | Lieve: 🞎  Grave: 🞎  Orario: |
|  | _______________  _______________  _______________  _______________ | _______________  _______________  _______________  _______________ | _______________  _______________  _______________  _______________ | _______________  _______________  _______________  _______________ | _______________  _______________  _______________  _______________ |
| **Venerdì**  Fattori scatenanti | Lieve: 🞎  Grave: 🞎  Orario: | Lieve: 🞎  Grave: 🞎  Orario: | Lieve: 🞎  Grave: 🞎  Orario: | Lieve: 🞎  Grave: 🞎  Orario: | Lieve: 🞎  Grave: 🞎  Orario: |
|  | _______________  _______________  _______________  _______________ | _______________  _______________  _______________  _______________ | _______________  _______________  _______________  _______________ | _______________  _______________  _______________  _______________ | _______________  _______________  _______________  _______________ |
| **Sabato**  Fattori scatenanti | Lieve: 🞎  Grave: 🞎  Orario: | Lieve: 🞎  Grave: 🞎  Orario: | Lieve: 🞎  Grave: 🞎  Orario: | Lieve: 🞎  Grave: 🞎  Orario: | Lieve: 🞎  Grave: 🞎  Orario: |
|  | _______________  _______________  _______________  _______________ | _______________  _______________  _______________  _______________ | _______________  _______________  _______________  _______________ | _______________  _______________  _______________  _______________ | _______________  _______________  _______________  _______________ |
| **Domenica**  Fattori scatenanti | Lieve: 🞎  Grave: 🞎  Orario: | Lieve: 🞎  Grave: 🞎  Orario: | Lieve: 🞎  Grave: 🞎  Orario: | Lieve: 🞎  Grave: 🞎  Orario: | Lieve: 🞎  Grave: 🞎  Orario: |
|  | _______________  _______________  _______________  _______________ | _______________  _______________  _______________  _______________ | _______________  _______________  _______________  _______________ | _______________  _______________  _______________  _______________ | _______________  _______________  _______________  _______________ |

| **Settimana**  **2** | **Insonnia o sonno**  **frammentato** | **Agitazione o ansia** | **Aggressività fisica o verbale** | **Attività motoria aberrante** | **Deliri o allucinazioni** |
| --- | --- | --- | --- | --- | --- |
| **Lunedì**  Fattori scatenanti | Lieve: 🞎  Grave: 🞎  Orario: | Lieve: 🞎  Grave: 🞎  Orario: | Lieve: 🞎  Grave: 🞎  Orario: | Lieve: 🞎  Grave: 🞎  Orario: | Lieve: 🞎  Grave: 🞎  Orario: |
|  | _______________  _______________  _______________  _______________ | _______________  _______________  _______________  _______________ | _______________  _______________  _______________  _______________ | _______________  _______________  _______________  _______________ | _______________  _______________  _______________  _______________ |
| **Martedì**  Fattori scatenanti | Lieve: 🞎  Grave: 🞎  Orario: | Lieve: 🞎  Grave: 🞎  Orario: | Lieve: 🞎  Grave: 🞎  Orario: | Lieve: 🞎  Grave: 🞎  Orario: | Lieve: 🞎  Grave: 🞎  Orario: |
|  | _______________  _______________  _______________  _______________ | _______________  _______________  _______________  _______________ | _______________  _______________  _______________  _______________ | _______________  _______________  _______________  _______________ | _______________  _______________  _______________  _______________ |
| **Mercoledì**  Fattori scatenanti | Lieve: 🞎  Grave: 🞎  Orario: | Lieve: 🞎  Grave: 🞎  Orario: | Lieve: 🞎  Grave: 🞎  Orario: | Lieve: 🞎  Grave: 🞎  Orario: | Lieve: 🞎  Grave: 🞎  Orario: |
|  | _______________  _______________  _______________  _______________ | _______________  _______________  _______________  _______________ | _______________  _______________  _______________  _______________ | _______________  _______________  _______________  _______________ | _______________  _______________  _______________  _______________ |
| **Giovedì**  Fattori scatenanti | Lieve: 🞎  Grave: 🞎  Orario: | Lieve: 🞎  Grave: 🞎  Orario: | Lieve: 🞎  Grave: 🞎  Orario: | Lieve: 🞎  Grave: 🞎  Orario: | Lieve: 🞎  Grave: 🞎  Orario: |
|  | _______________  _______________  _______________  _______________ | _______________  _______________  _______________  _______________ | _______________  _______________  _______________  _______________ | _______________  _______________  _______________  _______________ | _______________  _______________  _______________  _______________ |
| **Venerdì**  Fattori scatenanti | Lieve: 🞎  Grave: 🞎  Orario: | Lieve: 🞎  Grave: 🞎  Orario: | Lieve: 🞎  Grave: 🞎  Orario: | Lieve: 🞎  Grave: 🞎  Orario: | Lieve: 🞎  Grave: 🞎  Orario: |
|  | _______________  _______________  _______________  _______________ | _______________  _______________  _______________  _______________ | _______________  _______________  _______________  _______________ | _______________  _______________  _______________  _______________ | _______________  _______________  _______________  _______________ |
| **Sabato**  Fattori scatenanti | Lieve: 🞎  Grave: 🞎  Orario: | Lieve: 🞎  Grave: 🞎  Orario: | Lieve: 🞎  Grave: 🞎  Orario: | Lieve: 🞎  Grave: 🞎  Orario: | Lieve: 🞎  Grave: 🞎  Orario: |
|  | _______________  _______________  _______________  _______________ | _______________  _______________  _______________  _______________ | _______________  _______________  _______________  _______________ | _______________  _______________  _______________  _______________ | _______________  _______________  _______________  _______________ |
| **Domenica**  Fattori scatenanti | Lieve: 🞎  Grave: 🞎  Orario: | Lieve: 🞎  Grave: 🞎  Orario: | Lieve: 🞎  Grave: 🞎  Orario: | Lieve: 🞎  Grave: 🞎  Orario: | Lieve: 🞎  Grave: 🞎  Orario: |
|  | _______________  _______________  _______________  _______________ | _______________  _______________  _______________  _______________ | _______________  _______________  _______________  _______________ | _______________  _______________  _______________  _______________ | _______________  _______________  _______________  _______________ |

| **Settimana**  **3** | **Insonnia o sonno**  **frammentato** | **Agitazione o ansia** | **Aggressività fisica o verbale** | **Attività motoria aberrante** | **Deliri o allucinazioni** |
| --- | --- | --- | --- | --- | --- |
| **Lunedì**  Fattori scatenanti | Lieve: 🞎  Grave: 🞎  Orario: | Lieve: 🞎  Grave: 🞎  Orario: | Lieve: 🞎  Grave: 🞎  Orario: | Lieve: 🞎  Grave: 🞎  Orario: | Lieve: 🞎  Grave: 🞎  Orario: |
|  | _______________  _______________  _______________  _______________ | _______________  _______________  _______________  _______________ | _______________  _______________  _______________  _______________ | _______________  _______________  _______________  _______________ | _______________  _______________  _______________  _______________ |
| **Martedì**  Fattori scatenanti | Lieve: 🞎  Grave: 🞎  Orario: | Lieve: 🞎  Grave: 🞎  Orario: | Lieve: 🞎  Grave: 🞎  Orario: | Lieve: 🞎  Grave: 🞎  Orario: | Lieve: 🞎  Grave: 🞎  Orario: |
|  | _______________  _______________  _______________  _______________ | _______________  _______________  _______________  _______________ | _______________  _______________  _______________  _______________ | _______________  _______________  _______________  _______________ | _______________  _______________  _______________  _______________ |
| **Mercoledì**  Fattori scatenanti | Lieve: 🞎  Grave: 🞎  Orario: | Lieve: 🞎  Grave: 🞎  Orario: | Lieve: 🞎  Grave: 🞎  Orario: | Lieve: 🞎  Grave: 🞎  Orario: | Lieve: 🞎  Grave: 🞎  Orario: |
|  | _______________  _______________  _______________  _______________ | _______________  _______________  _______________  _______________ | _______________  _______________  _______________  _______________ | _______________  _______________  _______________  _______________ | _______________  _______________  _______________  _______________ |
| **Giovedì**  Fattori scatenanti | Lieve: 🞎  Grave: 🞎  Orario: | Lieve: 🞎  Grave: 🞎  Orario: | Lieve: 🞎  Grave: 🞎  Orario: | Lieve: 🞎  Grave: 🞎  Orario: | Lieve: 🞎  Grave: 🞎  Orario: |
|  | _______________  _______________  _______________  _______________ | _______________  _______________  _______________  _______________ | _______________  _______________  _______________  _______________ | _______________  _______________  _______________  _______________ | _______________  _______________  _______________  _______________ |
| **Venerdì**  Fattori scatenanti | Lieve: 🞎  Grave: 🞎  Orario: | Lieve: 🞎  Grave: 🞎  Orario: | Lieve: 🞎  Grave: 🞎  Orario: | Lieve: 🞎  Grave: 🞎  Orario: | Lieve: 🞎  Grave: 🞎  Orario: |
|  | _______________  _______________  _______________  _______________ | _______________  _______________  _______________  _______________ | _______________  _______________  _______________  _______________ | _______________  _______________  _______________  _______________ | _______________  _______________  _______________  _______________ |
| **Sabato**  Fattori scatenanti | Lieve: 🞎  Grave: 🞎  Orario: | Lieve: 🞎  Grave: 🞎  Orario: | Lieve: 🞎  Grave: 🞎  Orario: | Lieve: 🞎  Grave: 🞎  Orario: | Lieve: 🞎  Grave: 🞎  Orario: |
|  | _______________  _______________  _______________  _______________ | _______________  _______________  _______________  _______________ | _______________  _______________  _______________  _______________ | _______________  _______________  _______________  _______________ | _______________  _______________  _______________  _______________ |
| **Domenica**  Fattori scatenanti | Lieve: 🞎  Grave: 🞎  Orario: | Lieve: 🞎  Grave: 🞎  Orario: | Lieve: 🞎  Grave: 🞎  Orario: | Lieve: 🞎  Grave: 🞎  Orario: | Lieve: 🞎  Grave: 🞎  Orario: |
|  | _______________  _______________  _______________  _______________ | _______________  _______________  _______________  _______________ | _______________  _______________  _______________  _______________ | _______________  _______________  _______________  _______________ | _______________  _______________  _______________  _______________ |

| **Settimana**  **4** | **Insonnia o sonno**  **frammentato** | **Agitazione o ansia** | **Aggressività fisica o verbale** | **Attività motoria aberrante** | **Deliri o allucinazioni** |
| --- | --- | --- | --- | --- | --- |
| **Lunedì**  Fattori scatenanti | Lieve: 🞎  Grave: 🞎  Orario: | Lieve: 🞎  Grave: 🞎  Orario: | Lieve: 🞎  Grave: 🞎  Orario: | Lieve: 🞎  Grave: 🞎  Orario: | Lieve: 🞎  Grave: 🞎  Orario: |
|  | _______________  _______________  _______________  _______________ | _______________  _______________  _______________  _______________ | _______________  _______________  _______________  _______________ | _______________  _______________  _______________  _______________ | _______________  _______________  _______________  _______________ |
| **Martedì**  Fattori scatenanti | Lieve: 🞎  Grave: 🞎  Orario: | Lieve: 🞎  Grave: 🞎  Orario: | Lieve: 🞎  Grave: 🞎  Orario: | Lieve: 🞎  Grave: 🞎  Orario: | Lieve: 🞎  Grave: 🞎  Orario: |
|  | _______________  _______________  _______________  _______________ | _______________  _______________  _______________  _______________ | _______________  _______________  _______________  _______________ | _______________  _______________  _______________  _______________ | _______________  _______________  _______________  _______________ |
| **Mercoledì**  Fattori scatenanti | Lieve: 🞎  Grave: 🞎  Orario: | Lieve: 🞎  Grave: 🞎  Orario: | Lieve: 🞎  Grave: 🞎  Orario: | Lieve: 🞎  Grave: 🞎  Orario: | Lieve: 🞎  Grave: 🞎  Orario: |
|  | _______________  _______________  _______________  _______________ | _______________  _______________  _______________  _______________ | _______________  _______________  _______________  _______________ | _______________  _______________  _______________  _______________ | _______________  _______________  _______________  _______________ |
| **Giovedì**  Fattori scatenanti | Lieve: 🞎  Grave: 🞎  Orario: | Lieve: 🞎  Grave: 🞎  Orario: | Lieve: 🞎  Grave: 🞎  Orario: | Lieve: 🞎  Grave: 🞎  Orario: | Lieve: 🞎  Grave: 🞎  Orario: |
|  | _______________  _______________  _______________  _______________ | _______________  _______________  _______________  _______________ | _______________  _______________  _______________  _______________ | _______________  _______________  _______________  _______________ | _______________  _______________  _______________  _______________ |
| **Venerdì**  Fattori scatenanti | Lieve: 🞎  Grave: 🞎  Orario: | Lieve: 🞎  Grave: 🞎  Orario: | Lieve: 🞎  Grave: 🞎  Orario: | Lieve: 🞎  Grave: 🞎  Orario: | Lieve: 🞎  Grave: 🞎  Orario: |
|  | _______________  _______________  _______________  _______________ | _______________  _______________  _______________  _______________ | _______________  _______________  _______________  _______________ | _______________  _______________  _______________  _______________ | _______________  _______________  _______________  _______________ |
| **Sabato**  Fattori scatenanti | Lieve: 🞎  Grave: 🞎  Orario: | Lieve: 🞎  Grave: 🞎  Orario: | Lieve: 🞎  Grave: 🞎  Orario: | Lieve: 🞎  Grave: 🞎  Orario: | Lieve: 🞎  Grave: 🞎  Orario: |
|  | _______________  _______________  _______________  _______________ | _______________  _______________  _______________  _______________ | _______________  _______________  _______________  _______________ | _______________  _______________  _______________  _______________ | _______________  _______________  _______________  _______________ |
| **Domenica**  Fattori scatenanti | Lieve: 🞎  Grave: 🞎  Orario: | Lieve: 🞎  Grave: 🞎  Orario: | Lieve: 🞎  Grave: 🞎  Orario: | Lieve: 🞎  Grave: 🞎  Orario: | Lieve: 🞎  Grave: 🞎  Orario: |
|  | _______________  _______________  _______________  _______________ | _______________  _______________  _______________  _______________ | _______________  _______________  _______________  _______________ | _______________  _______________  _______________  _______________ | _______________  _______________  _______________  _______________ |

| **Settimana**  **5** | **Insonnia o sonno**  **frammentato** | **Agitazione o ansia** | **Aggressività fisica o verbale** | **Attività motoria aberrante** | **Deliri o allucinazioni** |
| --- | --- | --- | --- | --- | --- |
| **Lunedì**  Fattori scatenanti | Lieve: 🞎  Grave: 🞎  Orario: | Lieve: 🞎  Grave: 🞎  Orario: | Lieve: 🞎  Grave: 🞎  Orario: | Lieve: 🞎  Grave: 🞎  Orario: | Lieve: 🞎  Grave: 🞎  Orario: |
|  | _______________  _______________  _______________  _______________ | _______________  _______________  _______________  _______________ | _______________  _______________  _______________  _______________ | _______________  _______________  _______________  _______________ | _______________  _______________  _______________  _______________ |
| **Martedì**  Fattori scatenanti | Lieve: 🞎  Grave: 🞎  Orario: | Lieve: 🞎  Grave: 🞎  Orario: | Lieve: 🞎  Grave: 🞎  Orario: | Lieve: 🞎  Grave: 🞎  Orario: | Lieve: 🞎  Grave: 🞎  Orario: |
|  | _______________  _______________  _______________  _______________ | _______________  _______________  _______________  _______________ | _______________  _______________  _______________  _______________ | _______________  _______________  _______________  _______________ | _______________  _______________  _______________  _______________ |
| **Mercoledì**  Fattori scatenanti | Lieve: 🞎  Grave: 🞎  Orario: | Lieve: 🞎  Grave: 🞎  Orario: | Lieve: 🞎  Grave: 🞎  Orario: | Lieve: 🞎  Grave: 🞎  Orario: | Lieve: 🞎  Grave: 🞎  Orario: |
|  | _______________  _______________  _______________  _______________ | _______________  _______________  _______________  _______________ | _______________  _______________  _______________  _______________ | _______________  _______________  _______________  _______________ | _______________  _______________  _______________  _______________ |
| **Giovedì**  Fattori scatenanti | Lieve: 🞎  Grave: 🞎  Orario: | Lieve: 🞎  Grave: 🞎  Orario: | Lieve: 🞎  Grave: 🞎  Orario: | Lieve: 🞎  Grave: 🞎  Orario: | Lieve: 🞎  Grave: 🞎  Orario: |
|  | _______________  _______________  _______________  _______________ | _______________  _______________  _______________  _______________ | _______________  _______________  _______________  _______________ | _______________  _______________  _______________  _______________ | _______________  _______________  _______________  _______________ |
| **Venerdì**  Fattori scatenanti | Lieve: 🞎  Grave: 🞎  Orario: | Lieve: 🞎  Grave: 🞎  Orario: | Lieve: 🞎  Grave: 🞎  Orario: | Lieve: 🞎  Grave: 🞎  Orario: | Lieve: 🞎  Grave: 🞎  Orario: |
|  | _______________  _______________  _______________  _______________ | _______________  _______________  _______________  _______________ | _______________  _______________  _______________  _______________ | _______________  _______________  _______________  _______________ | _______________  _______________  _______________  _______________ |
| **Sabato**  Fattori scatenanti | Lieve: 🞎  Grave: 🞎  Orario: | Lieve: 🞎  Grave: 🞎  Orario: | Lieve: 🞎  Grave: 🞎  Orario: | Lieve: 🞎  Grave: 🞎  Orario: | Lieve: 🞎  Grave: 🞎  Orario: |
|  | _______________  _______________  _______________  _______________ | _______________  _______________  _______________  _______________ | _______________  _______________  _______________  _______________ | _______________  _______________  _______________  _______________ | _______________  _______________  _______________  _______________ |
| **Domenica**  Fattori scatenanti | Lieve: 🞎  Grave: 🞎  Orario: | Lieve: 🞎  Grave: 🞎  Orario: | Lieve: 🞎  Grave: 🞎  Orario: | Lieve: 🞎  Grave: 🞎  Orario: | Lieve: 🞎  Grave: 🞎  Orario: |
|  | _______________  _______________  _______________  _______________ | _______________  _______________  _______________  _______________ | _______________  _______________  _______________  _______________ | _______________  _______________  _______________  _______________ | _______________  _______________  _______________  _______________ |

| **Settimana**  **6** | **Insonnia o sonno**  **frammentato** | **Agitazione o ansia** | **Aggressività fisica o verbale** | **Attività motoria aberrante** | **Deliri o allucinazioni** |
| --- | --- | --- | --- | --- | --- |
| **Lunedì**  Fattori scatenanti | Lieve: 🞎  Grave: 🞎  Orario: | Lieve: 🞎  Grave: 🞎  Orario: | Lieve: 🞎  Grave: 🞎  Orario: | Lieve: 🞎  Grave: 🞎  Orario: | Lieve: 🞎  Grave: 🞎  Orario: |
|  | _______________  _______________  _______________  _______________ | _______________  _______________  _______________  _______________ | _______________  _______________  _______________  _______________ | _______________  _______________  _______________  _______________ | _______________  _______________  _______________  _______________ |
| **Martedì**  Fattori scatenanti | Lieve: 🞎  Grave: 🞎  Orario: | Lieve: 🞎  Grave: 🞎  Orario: | Lieve: 🞎  Grave: 🞎  Orario: | Lieve: 🞎  Grave: 🞎  Orario: | Lieve: 🞎  Grave: 🞎  Orario: |
|  | _______________  _______________  _______________  _______________ | _______________  _______________  _______________  _______________ | _______________  _______________  _______________  _______________ | _______________  _______________  _______________  _______________ | _______________  _______________  _______________  _______________ |
| **Mercoledì**  Fattori scatenanti | Lieve: 🞎  Grave: 🞎  Orario: | Lieve: 🞎  Grave: 🞎  Orario: | Lieve: 🞎  Grave: 🞎  Orario: | Lieve: 🞎  Grave: 🞎  Orario: | Lieve: 🞎  Grave: 🞎  Orario: |
|  | _______________  _______________  _______________  _______________ | _______________  _______________  _______________  _______________ | _______________  _______________  _______________  _______________ | _______________  _______________  _______________  _______________ | _______________  _______________  _______________  _______________ |
| **Giovedì**  Fattori scatenanti | Lieve: 🞎  Grave: 🞎  Orario: | Lieve: 🞎  Grave: 🞎  Orario: | Lieve: 🞎  Grave: 🞎  Orario: | Lieve: 🞎  Grave: 🞎  Orario: | Lieve: 🞎  Grave: 🞎  Orario: |
|  | _______________  _______________  _______________  _______________ | _______________  _______________  _______________  _______________ | _______________  _______________  _______________  _______________ | _______________  _______________  _______________  _______________ | _______________  _______________  _______________  _______________ |
| **Venerdì**  Fattori scatenanti | Lieve: 🞎  Grave: 🞎  Orario: | Lieve: 🞎  Grave: 🞎  Orario: | Lieve: 🞎  Grave: 🞎  Orario: | Lieve: 🞎  Grave: 🞎  Orario: | Lieve: 🞎  Grave: 🞎  Orario: |
|  | _______________  _______________  _______________  _______________ | _______________  _______________  _______________  _______________ | _______________  _______________  _______________  _______________ | _______________  _______________  _______________  _______________ | _______________  _______________  _______________  _______________ |
| **Sabato**  Fattori scatenanti | Lieve: 🞎  Grave: 🞎  Orario: | Lieve: 🞎  Grave: 🞎  Orario: | Lieve: 🞎  Grave: 🞎  Orario: | Lieve: 🞎  Grave: 🞎  Orario: | Lieve: 🞎  Grave: 🞎  Orario: |
|  | _______________  _______________  _______________  _______________ | _______________  _______________  _______________  _______________ | _______________  _______________  _______________  _______________ | _______________  _______________  _______________  _______________ | _______________  _______________  _______________  _______________ |
| **Domenica**  Fattori scatenanti | Lieve: 🞎  Grave: 🞎  Orario: | Lieve: 🞎  Grave: 🞎  Orario: | Lieve: 🞎  Grave: 🞎  Orario: | Lieve: 🞎  Grave: 🞎  Orario: | Lieve: 🞎  Grave: 🞎  Orario: |
|  | _______________  _______________  _______________  _______________ | _______________  _______________  _______________  _______________ | _______________  _______________  _______________  _______________ | _______________  _______________  _______________  _______________ | _______________  _______________  _______________  _______________ |

| **Settimana**  **7** | **Insonnia o sonno**  **frammentato** | **Agitazione o ansia** | **Aggressività fisica o verbale** | **Attività motoria aberrante** | **Deliri o allucinazioni** |
| --- | --- | --- | --- | --- | --- |
| **Lunedì**  Fattori scatenanti | Lieve: 🞎  Grave: 🞎  Orario: | Lieve: 🞎  Grave: 🞎  Orario: | Lieve: 🞎  Grave: 🞎  Orario: | Lieve: 🞎  Grave: 🞎  Orario: | Lieve: 🞎  Grave: 🞎  Orario: |
|  | _______________  _______________  _______________  _______________ | _______________  _______________  _______________  _______________ | _______________  _______________  _______________  _______________ | _______________  _______________  _______________  _______________ | _______________  _______________  _______________  _______________ |
| **Martedì**  Fattori scatenanti | Lieve: 🞎  Grave: 🞎  Orario: | Lieve: 🞎  Grave: 🞎  Orario: | Lieve: 🞎  Grave: 🞎  Orario: | Lieve: 🞎  Grave: 🞎  Orario: | Lieve: 🞎  Grave: 🞎  Orario: |
|  | _______________  _______________  _______________  _______________ | _______________  _______________  _______________  _______________ | _______________  _______________  _______________  _______________ | _______________  _______________  _______________  _______________ | _______________  _______________  _______________  _______________ |
| **Mercoledì**  Fattori scatenanti | Lieve: 🞎  Grave: 🞎  Orario: | Lieve: 🞎  Grave: 🞎  Orario: | Lieve: 🞎  Grave: 🞎  Orario: | Lieve: 🞎  Grave: 🞎  Orario: | Lieve: 🞎  Grave: 🞎  Orario: |
|  | _______________  _______________  _______________  _______________ | _______________  _______________  _______________  _______________ | _______________  _______________  _______________  _______________ | _______________  _______________  _______________  _______________ | _______________  _______________  _______________  _______________ |
| **Giovedì**  Fattori scatenanti | Lieve: 🞎  Grave: 🞎  Orario: | Lieve: 🞎  Grave: 🞎  Orario: | Lieve: 🞎  Grave: 🞎  Orario: | Lieve: 🞎  Grave: 🞎  Orario: | Lieve: 🞎  Grave: 🞎  Orario: |
|  | _______________  _______________  _______________  _______________ | _______________  _______________  _______________  _______________ | _______________  _______________  _______________  _______________ | _______________  _______________  _______________  _______________ | _______________  _______________  _______________  _______________ |
| **Venerdì**  Fattori scatenanti | Lieve: 🞎  Grave: 🞎  Orario: | Lieve: 🞎  Grave: 🞎  Orario: | Lieve: 🞎  Grave: 🞎  Orario: | Lieve: 🞎  Grave: 🞎  Orario: | Lieve: 🞎  Grave: 🞎  Orario: |
|  | _______________  _______________  _______________  _______________ | _______________  _______________  _______________  _______________ | _______________  _______________  _______________  _______________ | _______________  _______________  _______________  _______________ | _______________  _______________  _______________  _______________ |
| **Sabato**  Fattori scatenanti | Lieve: 🞎  Grave: 🞎  Orario: | Lieve: 🞎  Grave: 🞎  Orario: | Lieve: 🞎  Grave: 🞎  Orario: | Lieve: 🞎  Grave: 🞎  Orario: | Lieve: 🞎  Grave: 🞎  Orario: |
|  | _______________  _______________  _______________  _______________ | _______________  _______________  _______________  _______________ | _______________  _______________  _______________  _______________ | _______________  _______________  _______________  _______________ | _______________  _______________  _______________  _______________ |
| **Domenica**  Fattori scatenanti | Lieve: 🞎  Grave: 🞎  Orario: | Lieve: 🞎  Grave: 🞎  Orario: | Lieve: 🞎  Grave: 🞎  Orario: | Lieve: 🞎  Grave: 🞎  Orario: | Lieve: 🞎  Grave: 🞎  Orario: |
|  | _______________  _______________  _______________  _______________ | _______________  _______________  _______________  _______________ | _______________  _______________  _______________  _______________ | _______________  _______________  _______________  _______________ | _______________  _______________  _______________  _______________ |

| **Settimana**  **8** | **Insonnia o sonno**  **frammentato** | **Agitazione o ansia** | **Aggressività fisica o verbale** | **Attività motoria aberrante** | **Deliri o allucinazioni** |
| --- | --- | --- | --- | --- | --- |
| **Lunedì**  Fattori scatenanti | Lieve: 🞎  Grave: 🞎  Orario: | Lieve: 🞎  Grave: 🞎  Orario: | Lieve: 🞎  Grave: 🞎  Orario: | Lieve: 🞎  Grave: 🞎  Orario: | Lieve: 🞎  Grave: 🞎  Orario: |
|  | _______________  _______________  _______________  _______________ | _______________  _______________  _______________  _______________ | _______________  _______________  _______________  _______________ | _______________  _______________  _______________  _______________ | _______________  _______________  _______________  _______________ |
| **Martedì**  Fattori scatenanti | Lieve: 🞎  Grave: 🞎  Orario: | Lieve: 🞎  Grave: 🞎  Orario: | Lieve: 🞎  Grave: 🞎  Orario: | Lieve: 🞎  Grave: 🞎  Orario: | Lieve: 🞎  Grave: 🞎  Orario: |
|  | _______________  _______________  _______________  _______________ | _______________  _______________  _______________  _______________ | _______________  _______________  _______________  _______________ | _______________  _______________  _______________  _______________ | _______________  _______________  _______________  _______________ |
| **Mercoledì**  Fattori scatenanti | Lieve: 🞎  Grave: 🞎  Orario: | Lieve: 🞎  Grave: 🞎  Orario: | Lieve: 🞎  Grave: 🞎  Orario: | Lieve: 🞎  Grave: 🞎  Orario: | Lieve: 🞎  Grave: 🞎  Orario: |
|  | _______________  _______________  _______________  _______________ | _______________  _______________  _______________  _______________ | _______________  _______________  _______________  _______________ | _______________  _______________  _______________  _______________ | _______________  _______________  _______________  _______________ |
| **Giovedì**  Fattori scatenanti | Lieve: 🞎  Grave: 🞎  Orario: | Lieve: 🞎  Grave: 🞎  Orario: | Lieve: 🞎  Grave: 🞎  Orario: | Lieve: 🞎  Grave: 🞎  Orario: | Lieve: 🞎  Grave: 🞎  Orario: |
|  | _______________  _______________  _______________  _______________ | _______________  _______________  _______________  _______________ | _______________  _______________  _______________  _______________ | _______________  _______________  _______________  _______________ | _______________  _______________  _______________  _______________ |
| **Venerdì**  Fattori scatenanti | Lieve: 🞎  Grave: 🞎  Orario: | Lieve: 🞎  Grave: 🞎  Orario: | Lieve: 🞎  Grave: 🞎  Orario: | Lieve: 🞎  Grave: 🞎  Orario: | Lieve: 🞎  Grave: 🞎  Orario: |
|  | _______________  _______________  _______________  _______________ | _______________  _______________  _______________  _______________ | _______________  _______________  _______________  _______________ | _______________  _______________  _______________  _______________ | _______________  _______________  _______________  _______________ |
| **Sabato**  Fattori scatenanti | Lieve: 🞎  Grave: 🞎  Orario: | Lieve: 🞎  Grave: 🞎  Orario: | Lieve: 🞎  Grave: 🞎  Orario: | Lieve: 🞎  Grave: 🞎  Orario: | Lieve: 🞎  Grave: 🞎  Orario: |
|  | _______________  _______________  _______________  _______________ | _______________  _______________  _______________  _______________ | _______________  _______________  _______________  _______________ | _______________  _______________  _______________  _______________ | _______________  _______________  _______________  _______________ |
| **Domenica**  Fattori scatenanti | Lieve: 🞎  Grave: 🞎  Orario: | Lieve: 🞎  Grave: 🞎  Orario: | Lieve: 🞎  Grave: 🞎  Orario: | Lieve: 🞎  Grave: 🞎  Orario: | Lieve: 🞎  Grave: 🞎  Orario: |
|  | _______________  _______________  _______________  _______________ | _______________  _______________  _______________  _______________ | _______________  _______________  _______________  _______________ | _______________  _______________  _______________  _______________ | _______________  _______________  _______________  _______________ |

| **Settimana**  **9** | **Insonnia o sonno**  **frammentato** | **Agitazione o ansia** | **Aggressività fisica o verbale** | **Attività motoria aberrante** | **Deliri o allucinazioni** |
| --- | --- | --- | --- | --- | --- |
| **Lunedì**  Fattori scatenanti | Lieve: 🞎  Grave: 🞎  Orario: | Lieve: 🞎  Grave: 🞎  Orario: | Lieve: 🞎  Grave: 🞎  Orario: | Lieve: 🞎  Grave: 🞎  Orario: | Lieve: 🞎  Grave: 🞎  Orario: |
|  | _______________  _______________  _______________  _______________ | _______________  _______________  _______________  _______________ | _______________  _______________  _______________  _______________ | _______________  _______________  _______________  _______________ | _______________  _______________  _______________  _______________ |
| **Martedì**  Fattori scatenanti | Lieve: 🞎  Grave: 🞎  Orario: | Lieve: 🞎  Grave: 🞎  Orario: | Lieve: 🞎  Grave: 🞎  Orario: | Lieve: 🞎  Grave: 🞎  Orario: | Lieve: 🞎  Grave: 🞎  Orario: |
|  | _______________  _______________  _______________  _______________ | _______________  _______________  _______________  _______________ | _______________  _______________  _______________  _______________ | _______________  _______________  _______________  _______________ | _______________  _______________  _______________  _______________ |
| **Mercoledì**  Fattori scatenanti | Lieve: 🞎  Grave: 🞎  Orario: | Lieve: 🞎  Grave: 🞎  Orario: | Lieve: 🞎  Grave: 🞎  Orario: | Lieve: 🞎  Grave: 🞎  Orario: | Lieve: 🞎  Grave: 🞎  Orario: |
|  | _______________  _______________  _______________  _______________ | _______________  _______________  _______________  _______________ | _______________  _______________  _______________  _______________ | _______________  _______________  _______________  _______________ | _______________  _______________  _______________  _______________ |
| **Giovedì**  Fattori scatenanti | Lieve: 🞎  Grave: 🞎  Orario: | Lieve: 🞎  Grave: 🞎  Orario: | Lieve: 🞎  Grave: 🞎  Orario: | Lieve: 🞎  Grave: 🞎  Orario: | Lieve: 🞎  Grave: 🞎  Orario: |
|  | _______________  _______________  _______________  _______________ | _______________  _______________  _______________  _______________ | _______________  _______________  _______________  _______________ | _______________  _______________  _______________  _______________ | _______________  _______________  _______________  _______________ |
| **Venerdì**  Fattori scatenanti | Lieve: 🞎  Grave: 🞎  Orario: | Lieve: 🞎  Grave: 🞎  Orario: | Lieve: 🞎  Grave: 🞎  Orario: | Lieve: 🞎  Grave: 🞎  Orario: | Lieve: 🞎  Grave: 🞎  Orario: |
|  | _______________  _______________  _______________  _______________ | _______________  _______________  _______________  _______________ | _______________  _______________  _______________  _______________ | _______________  _______________  _______________  _______________ | _______________  _______________  _______________  _______________ |
| **Sabato**  Fattori scatenanti | Lieve: 🞎  Grave: 🞎  Orario: | Lieve: 🞎  Grave: 🞎  Orario: | Lieve: 🞎  Grave: 🞎  Orario: | Lieve: 🞎  Grave: 🞎  Orario: | Lieve: 🞎  Grave: 🞎  Orario: |
|  | _______________  _______________  _______________  _______________ | _______________  _______________  _______________  _______________ | _______________  _______________  _______________  _______________ | _______________  _______________  _______________  _______________ | _______________  _______________  _______________  _______________ |
| **Domenica**  Fattori scatenanti | Lieve: 🞎  Grave: 🞎  Orario: | Lieve: 🞎  Grave: 🞎  Orario: | Lieve: 🞎  Grave: 🞎  Orario: | Lieve: 🞎  Grave: 🞎  Orario: | Lieve: 🞎  Grave: 🞎  Orario: |
|  | _______________  _______________  _______________  _______________ | _______________  _______________  _______________  _______________ | _______________  _______________  _______________  _______________ | _______________  _______________  _______________  _______________ | _______________  _______________  _______________  _______________ |

| **Settimana**  **10** | **Insonnia o sonno**  **frammentato** | **Agitazione o ansia** | **Aggressività fisica o verbale** | **Attività motoria aberrante** | **Deliri o allucinazioni** |
| --- | --- | --- | --- | --- | --- |
| **Lunedì**  Fattori scatenanti | Lieve: 🞎  Grave: 🞎  Orario: | Lieve: 🞎  Grave: 🞎  Orario: | Lieve: 🞎  Grave: 🞎  Orario: | Lieve: 🞎  Grave: 🞎  Orario: | Lieve: 🞎  Grave: 🞎  Orario: |
|  | _______________  _______________  _______________  _______________ | _______________  _______________  _______________  _______________ | _______________  _______________  _______________  _______________ | _______________  _______________  _______________  _______________ | _______________  _______________  _______________  _______________ |
| **Martedì**  Fattori scatenanti | Lieve: 🞎  Grave: 🞎  Orario: | Lieve: 🞎  Grave: 🞎  Orario: | Lieve: 🞎  Grave: 🞎  Orario: | Lieve: 🞎  Grave: 🞎  Orario: | Lieve: 🞎  Grave: 🞎  Orario: |
|  | _______________  _______________  _______________  _______________ | _______________  _______________  _______________  _______________ | _______________  _______________  _______________  _______________ | _______________  _______________  _______________  _______________ | _______________  _______________  _______________  _______________ |
| **Mercoledì**  Fattori scatenanti | Lieve: 🞎  Grave: 🞎  Orario: | Lieve: 🞎  Grave: 🞎  Orario: | Lieve: 🞎  Grave: 🞎  Orario: | Lieve: 🞎  Grave: 🞎  Orario: | Lieve: 🞎  Grave: 🞎  Orario: |
|  | _______________  _______________  _______________  _______________ | _______________  _______________  _______________  _______________ | _______________  _______________  _______________  _______________ | _______________  _______________  _______________  _______________ | _______________  _______________  _______________  _______________ |
| **Giovedì**  Fattori scatenanti | Lieve: 🞎  Grave: 🞎  Orario: | Lieve: 🞎  Grave: 🞎  Orario: | Lieve: 🞎  Grave: 🞎  Orario: | Lieve: 🞎  Grave: 🞎  Orario: | Lieve: 🞎  Grave: 🞎  Orario: |
|  | _______________  _______________  _______________  _______________ | _______________  _______________  _______________  _______________ | _______________  _______________  _______________  _______________ | _______________  _______________  _______________  _______________ | _______________  _______________  _______________  _______________ |
| **Venerdì**  Fattori scatenanti | Lieve: 🞎  Grave: 🞎  Orario: | Lieve: 🞎  Grave: 🞎  Orario: | Lieve: 🞎  Grave: 🞎  Orario: | Lieve: 🞎  Grave: 🞎  Orario: | Lieve: 🞎  Grave: 🞎  Orario: |
|  | _______________  _______________  _______________  _______________ | _______________  _______________  _______________  _______________ | _______________  _______________  _______________  _______________ | _______________  _______________  _______________  _______________ | _______________  _______________  _______________  _______________ |
| **Sabato**  Fattori scatenanti | Lieve: 🞎  Grave: 🞎  Orario: | Lieve: 🞎  Grave: 🞎  Orario: | Lieve: 🞎  Grave: 🞎  Orario: | Lieve: 🞎  Grave: 🞎  Orario: | Lieve: 🞎  Grave: 🞎  Orario: |
|  | _______________  _______________  _______________  _______________ | _______________  _______________  _______________  _______________ | _______________  _______________  _______________  _______________ | _______________  _______________  _______________  _______________ | _______________  _______________  _______________  _______________ |
| **Domenica**  Fattori scatenanti | Lieve: 🞎  Grave: 🞎  Orario: | Lieve: 🞎  Grave: 🞎  Orario: | Lieve: 🞎  Grave: 🞎  Orario: | Lieve: 🞎  Grave: 🞎  Orario: | Lieve: 🞎  Grave: 🞎  Orario: |
|  | _______________  _______________  _______________  _______________ | _______________  _______________  _______________  _______________ | _______________  _______________  _______________  _______________ | _______________  _______________  _______________  _______________ | _______________  _______________  _______________  _______________ |

| **Settimana**  **11** | **Insonnia o sonno**  **frammentato** | **Agitazione o ansia** | **Aggressività fisica o verbale** | **Attività motoria aberrante** | **Deliri o allucinazioni** |
| --- | --- | --- | --- | --- | --- |
| **Lunedì**  Fattori scatenanti | Lieve: 🞎  Grave: 🞎  Orario: | Lieve: 🞎  Grave: 🞎  Orario: | Lieve: 🞎  Grave: 🞎  Orario: | Lieve: 🞎  Grave: 🞎  Orario: | Lieve: 🞎  Grave: 🞎  Orario: |
|  | _______________  _______________  _______________  _______________ | _______________  _______________  _______________  _______________ | _______________  _______________  _______________  _______________ | _______________  _______________  _______________  _______________ | _______________  _______________  _______________  _______________ |
| **Martedì**  Fattori scatenanti | Lieve: 🞎  Grave: 🞎  Orario: | Lieve: 🞎  Grave: 🞎  Orario: | Lieve: 🞎  Grave: 🞎  Orario: | Lieve: 🞎  Grave: 🞎  Orario: | Lieve: 🞎  Grave: 🞎  Orario: |
|  | _______________  _______________  _______________  _______________ | _______________  _______________  _______________  _______________ | _______________  _______________  _______________  _______________ | _______________  _______________  _______________  _______________ | _______________  _______________  _______________  _______________ |
| **Mercoledì**  Fattori scatenanti | Lieve: 🞎  Grave: 🞎  Orario: | Lieve: 🞎  Grave: 🞎  Orario: | Lieve: 🞎  Grave: 🞎  Orario: | Lieve: 🞎  Grave: 🞎  Orario: | Lieve: 🞎  Grave: 🞎  Orario: |
|  | _______________  _______________  _______________  _______________ | _______________  _______________  _______________  _______________ | _______________  _______________  _______________  _______________ | _______________  _______________  _______________  _______________ | _______________  _______________  _______________  _______________ |
| **Giovedì**  Fattori scatenanti | Lieve: 🞎  Grave: 🞎  Orario: | Lieve: 🞎  Grave: 🞎  Orario: | Lieve: 🞎  Grave: 🞎  Orario: | Lieve: 🞎  Grave: 🞎  Orario: | Lieve: 🞎  Grave: 🞎  Orario: |
|  | _______________  _______________  _______________  _______________ | _______________  _______________  _______________  _______________ | _______________  _______________  _______________  _______________ | _______________  _______________  _______________  _______________ | _______________  _______________  _______________  _______________ |
| **Venerdì**  Fattori scatenanti | Lieve: 🞎  Grave: 🞎  Orario: | Lieve: 🞎  Grave: 🞎  Orario: | Lieve: 🞎  Grave: 🞎  Orario: | Lieve: 🞎  Grave: 🞎  Orario: | Lieve: 🞎  Grave: 🞎  Orario: |
|  | _______________  _______________  _______________  _______________ | _______________  _______________  _______________  _______________ | _______________  _______________  _______________  _______________ | _______________  _______________  _______________  _______________ | _______________  _______________  _______________  _______________ |
| **Sabato**  Fattori scatenanti | Lieve: 🞎  Grave: 🞎  Orario: | Lieve: 🞎  Grave: 🞎  Orario: | Lieve: 🞎  Grave: 🞎  Orario: | Lieve: 🞎  Grave: 🞎  Orario: | Lieve: 🞎  Grave: 🞎  Orario: |
|  | _______________  _______________  _______________  _______________ | _______________  _______________  _______________  _______________ | _______________  _______________  _______________  _______________ | _______________  _______________  _______________  _______________ | _______________  _______________  _______________  _______________ |
| **Domenica**  Fattori scatenanti | Lieve: 🞎  Grave: 🞎  Orario: | Lieve: 🞎  Grave: 🞎  Orario: | Lieve: 🞎  Grave: 🞎  Orario: | Lieve: 🞎  Grave: 🞎  Orario: | Lieve: 🞎  Grave: 🞎  Orario: |
|  | _______________  _______________  _______________  _______________ | _______________  _______________  _______________  _______________ | _______________  _______________  _______________  _______________ | _______________  _______________  _______________  _______________ | _______________  _______________  _______________  _______________ |

| **Settimana**  **12** | **Insonnia o sonno**  **frammentato** | **Agitazione o ansia** | **Aggressività fisica o verbale** | **Attività motoria aberrante** | **Deliri o allucinazioni** |
| --- | --- | --- | --- | --- | --- |
| **Lunedì**  Fattori scatenanti | Lieve: 🞎  Grave: 🞎  Orario: | Lieve: 🞎  Grave: 🞎  Orario: | Lieve: 🞎  Grave: 🞎  Orario: | Lieve: 🞎  Grave: 🞎  Orario: | Lieve: 🞎  Grave: 🞎  Orario: |
|  | _______________  _______________  _______________  _______________ | _______________  _______________  _______________  _______________ | _______________  _______________  _______________  _______________ | _______________  _______________  _______________  _______________ | _______________  _______________  _______________  _______________ |
| **Martedì**  Fattori scatenanti | Lieve: 🞎  Grave: 🞎  Orario: | Lieve: 🞎  Grave: 🞎  Orario: | Lieve: 🞎  Grave: 🞎  Orario: | Lieve: 🞎  Grave: 🞎  Orario: | Lieve: 🞎  Grave: 🞎  Orario: |
|  | _______________  _______________  _______________  _______________ | _______________  _______________  _______________  _______________ | _______________  _______________  _______________  _______________ | _______________  _______________  _______________  _______________ | _______________  _______________  _______________  _______________ |
| **Mercoledì**  Fattori scatenanti | Lieve: 🞎  Grave: 🞎  Orario: | Lieve: 🞎  Grave: 🞎  Orario: | Lieve: 🞎  Grave: 🞎  Orario: | Lieve: 🞎  Grave: 🞎  Orario: | Lieve: 🞎  Grave: 🞎  Orario: |
|  | _______________  _______________  _______________  _______________ | _______________  _______________  _______________  _______________ | _______________  _______________  _______________  _______________ | _______________  _______________  _______________  _______________ | _______________  _______________  _______________  _______________ |
| **Giovedì**  Fattori scatenanti | Lieve: 🞎  Grave: 🞎  Orario: | Lieve: 🞎  Grave: 🞎  Orario: | Lieve: 🞎  Grave: 🞎  Orario: | Lieve: 🞎  Grave: 🞎  Orario: | Lieve: 🞎  Grave: 🞎  Orario: |
|  | _______________  _______________  _______________  _______________ | _______________  _______________  _______________  _______________ | _______________  _______________  _______________  _______________ | _______________  _______________  _______________  _______________ | _______________  _______________  _______________  _______________ |
| **Venerdì**  Fattori scatenanti | Lieve: 🞎  Grave: 🞎  Orario: | Lieve: 🞎  Grave: 🞎  Orario: | Lieve: 🞎  Grave: 🞎  Orario: | Lieve: 🞎  Grave: 🞎  Orario: | Lieve: 🞎  Grave: 🞎  Orario: |
|  | _______________  _______________  _______________  _______________ | _______________  _______________  _______________  _______________ | _______________  _______________  _______________  _______________ | _______________  _______________  _______________  _______________ | _______________  _______________  _______________  _______________ |
| **Sabato**  Fattori scatenanti | Lieve: 🞎  Grave: 🞎  Orario: | Lieve: 🞎  Grave: 🞎  Orario: | Lieve: 🞎  Grave: 🞎  Orario: | Lieve: 🞎  Grave: 🞎  Orario: | Lieve: 🞎  Grave: 🞎  Orario: |
|  | _______________  _______________  _______________  _______________ | _______________  _______________  _______________  _______________ | _______________  _______________  _______________  _______________ | _______________  _______________  _______________  _______________ | _______________  _______________  _______________  _______________ |
| **Domenica**  Fattori scatenanti | Lieve: 🞎  Grave: 🞎  Orario: | Lieve: 🞎  Grave: 🞎  Orario: | Lieve: 🞎  Grave: 🞎  Orario: | Lieve: 🞎  Grave: 🞎  Orario: | Lieve: 🞎  Grave: 🞎  Orario: |
|  | _______________  _______________  _______________  _______________ | _______________  _______________  _______________  _______________ | _______________  _______________  _______________  _______________ | _______________  _______________  _______________  _______________ | _______________  _______________  _______________  _______________ |
